# Spatially-resolved subtype progression reveals metabolic vulnerabilities in pancreatic ductal adenocarcinoma

**DOI:** 10.1186/s12943-026-02628-3

**Published:** 2026-03-27

**Authors:** Alexandra Maria Schmitt, Fabio Bennet Gätje, Michelle Yi Ran Tran, Teona Midelashvili, Xingbo Xu, Johannes Robert Fleischer, Henrik Spahn, Kelvin Lai, Tobias Tacke, Sara Younes, Anne Mittlmeier, Olaf Schulze, Kaida Hasanovic, Moritz Leander Blume, Sara Marie Rotter, Theon Robin Tesch, Carolin Schneider, Jovan Todorovic, Hanibal Bohnenberger, Stefan Kueffer, Philipp Ströbel, Argyris Papantonis, Ulrich Sax, David Agorku, Silvia Rüberg, Fabio El Yassouri, Rajeshwaran Purushothaman, Svenja Warratz, Nathalia Ferreira, Günter Schneider, Sebastian Igelmann, Melanie Planque, Sarah-Maria Fendt, Michael Ghadimi, Elisabeth Hessmann, Lutz Ackermann, Tiago De Oliveira, Lena-Christin Conradi

**Affiliations:** 1 Department of General, Visceral and Pediatric Surgery, University Medical Center Göttingen, Göttingen, Germany; 2 Clinical Research Unit 5002 (CRU5002), University Medical Center Göttingen, Göttingen, Germany; 3 Department of Cardiology and Pneumology, University Medical Center Göttingen, Göttingen, Germany; 4 German Center for Cardiovascular Research (DZHK), Partner Site Göttingen, Göttingen, Germany; 5 Eye Center, Medical Center, Faculty of Medicine, University of Freiburg, Freiburg, Germany; 6 Department of Pathology, University Medical Center Göttingen, Göttingen, Germany; 7 CCC-N (Comprehensive Cancer Center Lower Saxony), Göttingen, Germany; 8 Department of Medical Informatics, University Medical Center Göttingen, Göttingen, Germany; 9 Campus Institute Data Science, Georg August-University Göttingen, Göttingen, Germany; 10 Miltenyi Biotec B.V. & Co. KG, Bergisch Gladbach, Germany; 11 Institute of Organic and Biomolecular Chemistry, Göttingen, Germany; 12 Translational Molecular Imaging, Max-Planck-Institute for Multidisciplinary Sciences, Göttingen, Germany; 13 Laboratory of Cellular Metabolism and Metabolic Regulation, VIB Center for Cancer Biology, VIB, Leuven, Belgium; 14 Department of Oncology, Laboratory of Cellular Metabolism and Metabolic Regulation, KU Leuven and Leuven Cancer Institute (LKI), Leuven, Belgium; 15 Spatial Metabolomics Expertise Center, VIB Center for Cancer Biology, VIB, Leuven, Belgium; 16 Clinic of Gastroenterology, Gastrointestinal Oncology and Endocrinology, University Medical Center Göttingen, Göttingen, Germany

**Keywords:** Pancreatic ductal adenocarcinoma, Spatial transcriptomics, Classical and basal subtypes, Metabolism, Glycolysis, PFKFB3, Classical-to-basal trajectory, Subtype-agnostic targets

## Abstract

**Background:**

Pancreatic ductal adenocarcinoma (PDAC) exhibits profound molecular heterogeneity and poor prognosis, necessitating novel tailored therapies. The basal and classical subtypes - driven by glycolysis versus lipid metabolism - have distinct prognostic implications warranting further characterization of their underlying transcriptional mechanisms.

**Methods:**

Using spatial RNA sequencing we mapped PDAC molecular subtype heterogeneity, capturing spatially-resolved gene expression signatures and generating a comprehensive high-resolution dataset of 42,035 spatial spots. Subtype assignments were validated via multiplex immunofluorescence and quantitative analyses in patient-derived organoids and xenografts.

**Results:**

Our analysis resolved cancer cell signatures, deconvoluted intra-tumoral heterogeneity, and delineated an evolutional classical-to-basal trajectory. We identified metabolically ‘hot’, high-grade tumor niches characterized by concurrent enrichment of glycolysis and lipogenesis across both subtypes, nominating them as subtype-agnostic therapeutic targets. Preclinical models demonstrated that despite the basal subtype’s glycolysis dependence, both classical and basal tumors are susceptible to glycolysis inhibition.

**Conclusion:**

This work demonstrates that metabolic identity, spatial context, and tumor–stroma crosstalk are an inseparable triad that drives PDAC behavior. Our findings show that aggressive metabolic tumor niches can be targeted by glycolysis inhibition in a subtype-agnostic manner, challenging the dogma of subtype-specific therapeutic silos and highlighting highly adaptable energetic niches as reservoirs that drive tumor progression.

**Supplementary Information:**

The online version contains supplementary material available at 10.1186/s12943-026-02628-3.

## Introduction

Despite substantial advances in unraveling the molecular and genetic complexities of pancreatic cancer (PC), its 5-year survival rate remains around 13%, highlighting its intricate nature and substantial clinical challenges [1–4]. Projections indicate that PC could become the second leading cause of cancer-related deaths in the United States by 2030, underscoring the urgency for innovative treatment strategies [5]. Additionally, surgical resection, the only potential curative option, is often restricted by late diagnosis, rapid local expansion, and early metastasis, resulting in overall resection rates as low as 15–20% [6, 7].

Previously regarded as a single disease entity, PC and specifically pancreatic ductal adenocarcinoma (PDAC) have now been shown to exhibit significant heterogeneity, necessitating the development of tailored therapeutic approaches. Moreover, proper classification of cancer subtypes is crucial for aligning treatments with individual tumor characteristics, thereby optimizing clinical outcomes [8, 9]. Among the transcriptional subtypes identified in PDAC research, the most prevalent are the classical subtype, known for its glandular morphology and slightly improved sensitivity to chemotherapy, and the basal subtype, notorious for its squamous morphology, aggressiveness, and poor differentiation [10–16].

Abnormal metabolism, recognized as a hallmark of cancer [17], has become a key point in understanding PDAC. Pancreatic cancer exhibits significant metabolic plasticity [18, 19], which contributes to treatment resistance and adversely impacts patients’ outcome [20–23]. Therefore, to explore the metabolic profiles associated with different PDAC subtypes and their role in tumor heterogeneity and drug resistance, we conducted spatial RNA sequencing (spaRNA-seq) analyses on tissue samples from 14 PDAC patients. This approach allows metabolic programs to be examined in their native tissue context, within the desmoplastic stroma that shapes tumor cell states.

Consistent with this spatially constrained metabolic view, recent spatial and single-cell transcriptomic studies have shown that PDAC molecular subtypes are not uniform states but coexist and intermix within individual tumors, reflecting pronounced cellular plasticity and subtype-specific interactions with stromal, immune, vascular, and neural compartments [24–29]. Despite these insights, the functional relevance of such spatially-resolved tumor states, particularly with respect to targetable metabolic vulnerabilities, remains poorly defined and represents an important unmet need.

The comprehensive spaRNA-seq dataset generated in this study was validated through multiplexed-immunofluorescence staining and corroborated by parallel analyses on corresponding patient-derived organoids (PDOs). Diverging from marker-defined stratification [30–32], our analysis leverages spatially-resolved gene signatures to deconvolute tumor heterogeneity, uncovering its molecular trajectory and highlighting glycolysis as a critical metabolic divergence driving PDAC aggressiveness. Pharmacological inhibition of the glycolysis activator PFKFB3 with the novel small-molecule inhibitor KAN0438757 induces a metabolic shift in PDOs towards a classical-like state. Strikingly, this reprogramming sensitized PDOs to gemcitabine. In vivo validation in subtype-defined PDAC xenografts demonstrated that KAN0438757 combination therapy reduced tumor growth in both basal and classical tumors. These findings position glycolysis inhibition as a subtype-agnostic strategy to counteract therapeutic resistance.

By bridging spatial transcriptomics analyses, tumor heterogeneity, metabolic reprogramming, and preclinical validation, this work challenges the dogma of subtype-specific therapeutic silos. Targeting glycolysis alongside chemotherapy emerges as a paradigm to dismantle PDAC’s adaptive machinery, offering a clinically actionable path to improve survival in this molecularly complex malignancy.

## Methods

### Spatial transcriptomics

#### Slide preparation, staining, imaging

The Visium Spatial Gene Expression for FFPE Kit (10X Genomics, PN-1000338) was used to generate sequencing libraries. Prior to section placement, tissue adhesion was assured using the Visium Tissue Section Test Slides (10X Genomics, PN-1000347) and RNA-quality of FFPE blocks was assessed using RNeasy FFPE Kit (Qiagen, #73504) for RNA extraction and Agilent 2100 Bioanalyzer system with Agilent RNA 6000 Nano Kit (5067 − 1511) for determination of DV200. All samples reached DV200 of at least 50%. Sections of 5 μm thickness were cut from FFPE PDAC cancer tissue from 14 patients and placed on the capture areas of the Visium Spatial Gene Expression Slide (10X Genomics, PN-2000233). Each capture area with a size of 6.5 × 6.5 mm contains roughly 5,000 unique gene expression spots with a diameter of 55 μm. Following the manufacturers’ protocol, the sections were deparaffinized, H&E-stained, cover-slipped, and imaged at 40x magnification with the Glissando Objective Imaging scanner. Tissue permeabilization and construction of sequencing libraries was performed following the manufacturers’ protocol (10X Genomics, PN-1000338).

### Reverse transcription, spatial library preparation and sequencing

Libraries were sequenced using the DNBSEQ™ technology (BGI). Therefore, DNA Nanoballs (DNB) were created, and all samples were loaded on one flow cell using the DNBSEQ-G400 High-throughput Sequencing Set (BGI, 1000016970). Two samples were pooled together on one sequencing lane. The MGISEQ-2000 sequencer (BGI) was used with the following settings: Paired-end run, with 28 cycles for read1 (encoding spatial Barcode and UMI), 50 cycles for read2 (encoding the ligated probe insert), 10 cycles for i5 index and 10 cycles for i7 index (identifying each sample) (PE28 + 50 + 10 + 10). The sequencing depth was 300 Mio reads per lane, which equals a sequencing depth of 150 Mio reads per sample.

### Spatial transcriptomics data processing

After sequencing, libraries were de-multiplexed, mapped to the human transcriptome, and aligned to overlaying H&E images using SpaceRanger software (10X Genomics) and the manual alignment tool (LoupeBrowser v5.1.0, 10X Genomics). All further steps were performed using the UniApp (Unicle Biomedical Data Science, Belgium, https://unicle.com/), a commercially licensed web-based bioinformatics platform (accessed March 2023–January 2026). Analyses were conducted using the modules data pretreatment, dimension reduction, clustering, cluster marker genes, differential analysis, gene set enrichment, feature heatmap, cell-cell interaction analysis, feature engineering, rule-based meta-analysis, rank-based meta-analysis, gene regulatory network and trajectory inference. Default parameters were used unless otherwise specified.

### Quality control

Across all patients, 44,739 tissue-covered spots were detected. Detailed quality metrics for every sample are shown in Table S1. For quality filtering, spots with an expression of less than 200 genes/spot, ambiguous expression of canonical marker genes, not matching morphological structures or location on folded tissue were excluded, resulting in 42,035 high-quality spots included for further analysis.

### Graph-based clustering of single samples and cluster annotation

For clustering the spots of each sample, data was auto-scaled, and dimensional reduction was performed using PCA. The first 30 principal components were visualized using UMAP-reduced data. Graph-based Louvain clustering was performed to cluster the spots according to their gene expression profile (clustering-resolution = 0.8, k-nearest neighbors = 10). Since every spot captures transcriptomics of several spatially overlaying cells, the predominating cell types in every cluster were determined using canonical marker genes. Clusters were further investigated by identifying the top 50 enriched marker genes for each determined cluster.

### UpSet plots and Jaccard similarity PCA

UpSet plots were generated using the R package “UpSetR” (v1.4.0). We calculated the similarity of marker gene sets using pair-wise Jaccard similarity coefficients, comparing all clusters against all clusters in each dataset separately. The Jaccard similarity coefficient is defined as the size of the intersection divided by the size of the union of sets:

$$J(A, B)=\vert{A}\cap{B}\vert\:\vert{A}\cup{B}\vert=\vert{A}\cap{B}\vert\:\vert{A}\vert+\vert{B}\vert-\vert{A}\vert\cap\vert{B}\vert$$

Where *J* is the Jaccard index and *A* and *B* are two sets of marker genes.

### Trajectory inference

Trajectory analysis was performed using SCORPIUS (version 1.0.9) [33] by using the top 2000 highly variable genes and the following parameters: k = 4, number of principal components = 3. For visualization of gene expression trends along the inferred trajectory, a weighted moving average approach was employed for LOESS regression with a regression span of 0.75.

### RankProduct-based meta-analysis (Rank-based meta-analysis)

We used a modification of the previously described product-based meta-analysis [34], which penalizes genes that are not consistently up-regulated across all comparisons. In this case, the most up-regulated genes received the highest rank number (top ranking up-regulated genes), and the most down-regulated genes received the lowest rank number. We combined the rank numbers for all genes in all comparisons by calculating their product to obtain a final list of ranked genes in which the gene with the largest product was ranked at rank one.

### Gene set enrichment analysis

We used GSEA as implemented in the fgsea package (version 1.26.0) [35, 36] to compare gene expression signatures between groups. Gene set analysis was performed using gene sets selected from the msigdbr package (version 7.5.1), a collection of expert-annotated gene sets. GSEA scores were calculated for sets with a minimum of five detected genes, and all other parameters were set to default.

### Differential gene expression analysis

Prior to differential gene expression analysis, we performed pseudobulk transformation for cancer cell clusters and cancer cell subclusters independently, using the AggregateExpression function from Seurat R package. Subsequently, differential expression analysis between cancer cell clusters of classical and basal subtyped patients was performed using limma [37], as described previously [38]. Furthermore, differential gene expression analysis between classical and basal cancer cell subclusters was performed. For visualization in pathway mapping, genes from Glycolysis/Gluconeogenesis (M11521) canonical pathway derived from the KEGG database and *PFKFB*s were chosen and log fold-change values (A) were scaled according to the following equation:

$$-0.5+\frac{1}{1+{e}^{10*-\left(A\right)}}$$

A color scale with expression − 0.5 to 0.5 was used for mapping.

### Correlation heatmap analysis

All correlation heatmaps were created using the heatmaply package (version 1.4.2) [39] based on cluster-averaged gene expression to account for cell-to-cell transcriptomic stochasticity. The input data consisted of normalized data that was filtered to include the top 2000 highly variable genes and was auto-scaled. The following settings were applied: distance, Euclidean; agglomeration, complete; squared distance, false; clustering location, row, column; clustering type, p-value; p-value threshold, 0.95; bootstrap number, 10,000; force positive, false. Data was auto-scaled for visualization.

### Gene regulatory network

Gene regulatory network inference was performed using GRNboost2 as implemented in the UniApp from the arboreto package (version 0.1.6). Highly variable genes were selected from expression data of pure cancer areas across all patients and the top 500 features were used for network construction. GRNboost2 was run with default parameters, considering all genes in the selected feature set as potential regulators without additional filtering. Therefore, inferred regulatory relationships represent expression-based associations and do not necessarily indicate direct transcription factor–mediated regulation.

Network post-processing focused on hub-centered subnetworks for *MLPH* and *PFKFB3*. Regulatory interactions were filtered using an importance score cutoff of 0.5 to retain high-confidence edges.

### Cell-cell communication analysis (CellPhoneDB)

Cell–cell communication analysis was performed using the CellPhoneDB framework implemented in the UniApp platform. The analysis was conducted on the normalized gene expression matrix using CellPhoneDB’s statistical method to identify significant ligand–receptor interactions between annotated cell types.

Four analyses were performed. In the first, fibroblasts were defined as “sender“ cells and cancer cells as “receiver” cells (pure clusters from all patients) (Fig. S3 K). The second and third analyses assessed cancer-to-stroma interactions, with basal or classical cancer cells as “sender” cells and fibroblasts as “receiver” cells, respectively (Fig 3E). The fourth analysis evaluated autocrine interactions within cancer subtypes, with classical or basal cancer cells as both “sender” and “receiver”, resulting in two graphical representations (Fig. S4B).

For all analyses, interactions were evaluated using 1,000 permutations, with *p* < 0.05 considered statistically significant, no minimum expression threshold applied (% of observations expressing the gene set, set to 0), and subsampling enabled with 14,010 observations per iteration, reported with three decimal places. Additional subsampling parameters for analyses based on the split cancer subtypes included 100 principal components and log1p transformation disabled. Interactions were directional where appropriate, and for all analyses, only interactions with a mean interaction value > 0.5 were retained.

### Gene set variation analysis

We used gene set variation analysis (GSVA), as implemented in the GSVA R package (version 2.2.0), to convert the gene-by-cell matrix to a gene set-by-cell matrix using the default settings [40]. The gene sets used for GSVA were MSigDB Hallmarks and KEGG metabolism pathways. After converting the matrix, differential expression analysis was performed, comparing gene set expression in untreated PDOs (reference group) vs. gene set expression in treated PDOs (40 µM KAN0438757 for 24 h for all PDOs (*n* = 8, including mixed), for classical PDOs (*n* = 3) separately and for basal PDOs separately (*n* = 3).

### Data visualization

The UniApp (Unicle Biomedical Data Science, Belgium) was used for data visualization, including t-SNE plots, spatial plotting of spots on the H&E slide, heatmaps, and dot plots. Heatmaps are based on cluster-averaged gene expression to account for cell-to-cell transcriptomic stochasticity. In all heatmaps, data was auto-scaled for visualization.

### In vitro functional assays

#### Primary PDAC patient-derived organoid culture

Culture protocols are adapted, based on Boj et al. [41].

Growth medium for PDO culture was prepared by combining 19 mL of organoid splitting medium Advanced DMEM/F12 (Gibco, 11320033) supplemented with 1x GlutaMAX (Gibco, 35050061), 1x HEPES (Gibco, 15630106), and 1 mL Primocin (1x, InvivoGen, ant-pm-05) with 25 mL of 2x hAFM Wnt3a-conditioned media (in-house), 5 mL of 10x R-spondin-conditioned media (in-house), 50 µL mNoggin (100 µg/mL, Peprotech, 25038100UG), 25 µL A83-01 (1 mM, Tocris, 2939), 50 µL Human Epidermal Growth Factor (hEGF; 500 µg/mL, Invitrogen, PIRP8661), 50 µL human Fibroblast Growth Factor-10 (hFGF-10; 100 mg/mL, Peprotech, 10026100UG), 50 µL Gastrin I (100 µM, Sigma, 10047-33-3), 125 µL N-acetylcysteine (500 mM, Sigma, 616-91-1), 500 µL Nicotinamide (1 M, Sigma, 98-92-0), 1 mL B-27 supplement (50x, Gibco, 17504001), and 100 µL Primocin (50 mg/mL, InvivoGen, ant-pm-05).

### PDAC organoid treatment and live-cell imaging

PDAC organoids were generated by seeding 4,000 cells per well into 96-well plates pre-coated with 50 µL of Matrigel (Corning). The plates were incubated at 37 °C in a humidified atmosphere with 5% CO2 for 4 days to allow organoid formation. After this initial incubation, the organoids used for live-cell imaging were treated with growth medium supplemented with KAN0438757 at a concentration of 40 µM (200 µL per well) for 48 h to detect cumulative effects on PDO growth and viability. Dimethyl sulfoxide (DMSO) served as the control. Following the initial treatment, the drug-containing medium was removed and replaced with fresh medium containing 40 µM KAN0438757 for an additional 48 h to ensure sustained drug exposure. Subsequently, the media were changed to normal growth medium without drug for the remaining experimental period.

Organoid growth and viability were monitored continuously using the Incucyte^®^ S3 Live-Cell Analysis System (Sartorius, Germany), which captured phase-contrast images every 6 h over a total experiment duration of 156 h. Each condition was tested in duplicate to ensure the reliability of the data.

### PDAC OrganoidNet analysis

Images from the Incucyte imaging system were analyzed using OrganoIDNet, a deep-learning-based tool [42]. A StarDist model (version 12) was trained with a custom dataset of manually annotated bright field images from the Incucyte system. This model segmented organoids in the images, which were then classified as healthy or unhealthy based on mean pixel intensity. Organoids were also categorized by size into five bins: Tiny, Small, Medium, Large, and Huge. Shape deviation from a perfect circle was assessed using Eccentricity, with a value of zero indicating a perfect circle.

#### PDO treatment for bulk transcriptomics analysis

PDAC organoids were generated by seeding 4,000 cells per Matrigel drop (50µL) into 12-well plates (Corning). The plates were incubated at 37 °C in a humidified atmosphere with 5% CO2 for 4 days to allow organoid formation. After this initial incubation, the organoids were treated with growth medium supplemented with KAN0438757 at a concentration of 25 µM for 24 h to capture acute primary drug effects on transcriptional level. DMSO served as the control. Three Matrigel drops containing PDOs were used for each condition.

### PDO and PDX bulk transcriptomics - sequencing

RNA was isolated with Quick-RNA miniprep (Zymo Research, #R1055), according to the manufacturer’s instructions. RNA quality was assessed by measuring the RNA integrity number (RIN) using a Fragment Analyzer HS Total RNA Kit (DNF-472-FR; Agilent Technologies). Library preparation for RNA-Seq was performed on the STAR Hamilton NGS automation platform using the Illumina Stranded mRNA Prep Kit (Cat. No. 20040534) together with the Illumina RNA UD Indexes Set A, Ligation with 96 Indexes (Cat. No. 20091646), starting from 50 ng of total RNA. The size range of the final cDNA libraries was determined with the SS NGS Fragment 1–6000 bp Kit on the Fragment Analyzer (average 340 bp). Accurate quantification of cDNA libraries was carried out using the DeNovix DS-Series System. Sequencing was performed at the NGS Integrative Genomics Core Unit, UMG, using the Illumina NovaSeq 6000. The raw reads were quality controlled using fastqc (RRID: SCR_014583) and checked for both microbial and mouse contamination using Kraken [15] (RRID: SCR_005484) and fastq screen (RRID: SCR_000141), respectively. For PDX samples, reads classified as mouse-derived were excluded prior to alignment, and only human reads were mapped to the reference genome. Next, the sequence reads were trimmed using trimmomatic (RRID: SCR_011848), mapped to the reference genome (Ensembl, genome assembly GRCh38.p13) using star (RRID: SCR_004463), and finally the features were quantified using htseq-count (RRID: SCR_011867). All results were gathered into a comprehensive report using multiqc (RRID: SCR_014982). Additional information about the sequencing can be found in the metadata spreadsheets of the Gene Expression Omnibus (GEO) repository.

#### Panel sequencing

Molecular characterization of PDAC models was performed at the Institute of Human Genetics, UMG, Germany and was conducted as previously described [43]. Variants were assessed according to [44, 45]. The utilization and genetic characterization of human PDAC data, samples, and models have been approved by the ethical review board of the UMG (11/5/17).

#### PDO co-cultures

PDOs were cultured in Matrigel (Corning, 356234) within 24-well plates and maintained at 37 °C in a 5% CO₂ atmosphere. Medium changes were performed every 2–3 days by adding 0.5 mL of warm growth medium (GM). Human umbilical vein endothelial cells (HUVECs) were seeded in 175T flasks pre-coated with 0.1% gelatin and expanded in 1:1 supplemented ECGM2 medium (PromoCell, C-22011) and M199 medium (Gibco, 11825015) supplemented with 20% fetal bovine serum (Gibco, 10099141), 1% L-Glutamine (Gibco, 25030081), ECGS/Heparin (PromoCell, C-30120), and Anti-Anti (Gibco, 15240062), with medium changes every 2–3 days. Primary human fibroblasts were cultured in DMEM (PAN Biotech, F0435) supplemented with 10% FBS and 4 mM L-Glutamine under the same conditions. PDAC PDO co-cultures were established by seeding PDOs, HUVECs, and fibroblasts within a hydrogel matrix prepared from 12 mg fibrinogen (Sigma, 9001-32-5), 1200 µL unsupplemented ECGM2 medium (PromoCell, C-22011), 10% Matrigel (Corning, 356234), and 1% Aprotinin and solidified with 20% thrombin (1.5 U/ml, Sigma, 9002-04-4). Co-cultures were maintained for 5 days with medium changes every 2–3 days using a co-culture growth medium (CGM) prepared by mixing GM and ECGM2 with 1% Aprotinin. Following the incubation period, co-cultures were treated with KAN0438757 at a concentration of 30 µM for 48 h, with DMSO (Sigma, 67-68-5) as a control. On day 7, supernatants were collected for subsequent secretome analysis.

#### Secretomics - protein isolation, digestion, and mass spectrometry analysis

Secreted proteins were isolated from the supernatant using trichloroacetic acid (TCA)/acetone precipitation. Briefly, 250 µL of each sample was mixed with pre-cooled 10% TCA in acetone (1:5, v/v) and incubated overnight at -20 °C. Precipitated proteins were pelleted by centrifugation (30 min, 4 °C, 17,000 × g), washed with pre-cooled acetone, and reconstituted in Laemmli buffer. Proteins were separated on 4–12% NuPAGE Novex Bis-Tris gels (Invitrogen), stained with Coomassie, and the protein bands were excised. The gel pieces were subjected to reduction with dithiothreitol, alkylation with iodoacetamide, and overnight trypsin digestion. Extracted peptides were dried using a Speedvac and stored at -20 °C. Peptide analysis was performed using a Vanquish Neo UHPLC system coupled to an Exploris 480 mass spectrometer (Thermo Fisher Scientific) with an EasySpray ion source. Peptides (150 ng) were dissolved in loading buffer (2% acetonitrile, 0.1% TFA), trapped on a C18 trapping column (Thermo Fisher), and separated on an Aurora C18 analytical column (IonOpticks) using a 49-min linear gradient of 5–34% acetonitrile/0.1% formic acid at 400 nL/min and 50 °C. Data-independent acquisition (DIA) was performed using a 30-variable window isolation scheme (m/z 350–1150). Two technical replicates were acquired per biological sample. Protein identification and quantification were performed using the Pulsar algorithm in Spectronaut (v18.5, Biognosys) with default settings. DIA data were searched against the UniProtKB Homo sapiens and Bos taurus reference proteomes (revision 08-2023) and 51 common contaminants. Protein quantification was based on the integration of up to six fragment ions per peptide and up to 10 peptides per protein. Both identification and quantification were filtered at a 1% false discovery rate (FDR) using a decoy database strategy.

### Histology

For H&E staining, FFPE samples of PDAC were cut in 2-µm slides, deparaffinized in xylol (Carl Roth, Karlsruhe, Germany) and further subjected to descending series of ethanol (Carl Roth) (from 100 to 70% ethanol) following standard H&E staining protocol (3 min Mayer’s hematoxylin (Merck, Darmstadt, Germany), 2 × 2 min wash in distilled water, 1 min eosin (Merck) incubation and final dehydration in increasing ethanol series (from 70 to 100% ethanol) and xylol (all Carl Roth). Last, coverslips were mounted with Permount medium (Fisher Scientific). Grading was performed by a trained pathologist following WHO guidelines, as adapted by Haeberle and Esposito 2019 [46].

### Multiplex imaging and image analysis

Eleven tissue samples were sent to Miltenyi (Bergisch Gladbach, Germany) for MACSima™ imaging cyclic staining (MICS) as previously described [47]. The 29 markers used can be found in Key Resources’ table. The images were afterwards imported to MACS iQ View (Miltenyi Biotec B.V. & Co. KG) for further analysis. For the visualization, each channel was adapted individually. After segmentation (done in adapted default mode to *“Constrained donut”* as advised by the developer for cytoplasm, *Min./Max. diameter* to 20/80 pixels and *separation force* reduced to 40% for nuclei detection), the expression data of a total number of 622,260 cells was exported for the following statistical analysis. The cancer cells in the graded areas were defined by cancer marker expression (MUC1 > 50) and low collagen-expression (Collagen I < 500). Final groups for analysis were G1-2 (G1 and G1-2 areas; *n* = 3,820), G2 (*n* = 16,552) and G2-3 (G2-3 and G3 areas; *n* = 2,763).

### Cell culture

Human PDAC cell lines representing basal and classical molecular subtypes were maintained under standard culture conditions at 37 °C in a humidified atmosphere containing 5% CO₂. Basal cell lines included BxPC-3, MIA PaCa-2, PANC-1, and PaTu-8988T, whereas the classical cohort comprised Capan-1, Capan-2, and HPAF-II. All cell lines were cultured in their recommended growth media (MIA PaCa-2, PANC-1, PaTu-8988T, HPAF-II - DMEM, Gibco; BxPC-3, Capan-1, Capan-2 - RPMI 1640, Gibco), supplemented with 10% fetal bovine serum (FBS, Capricorn), 1% L-glutamine, and 1% penicillin–streptomycin (both Gibco). Cells were passaged at 70–80% confluency using standard trypsinization procedures (0.25% Trypsin, Gibco, for 5–10 min incubation) to ensure exponential growth during experiments.

### Cell viability

Cell viability was determined using the CellTiter-Blue Cell Viability Assay (G8081, Promega, Madison, USA) according to the manufacturer’s protocol. Briefly, 10^4^ cancer cells were plated in a black 96-well plate with a clear bottom and treated on the following day with a total volume of 100 µL per well. 1 h before the desired time point, 10 µL of the CellTiter-Blue reagent was added to each well. After 1 h, the fluorescence was recorded at 560_ex_/590_em_ nm using a microplate spectrophotometer. The mean of the no-cells background control values was subtracted from the sample readouts, then viability was calculated relative to the vehicle-treated control cells. All conditions were performed in triplicate.

### xCELLigence

Cells (1 × 10^4^/100 µL) were seeded in 16-well plates, bottom-coated with golden microelectrodes, and incubated in the xCELLigence Real-Time Cell Analysis (RTCA) S16 instrument (both, Aligent) at 37 °C, 5% CO₂ conditions. Every condition was tested in triplicates, and the assay was performed three times. Conditions were compared by fitting a saturation curve in logarithm and predicting the 80 h time point, which was then compared using a *t-test*. Shortly, by measuring the impedance of bottom-coated plates with microelectrodes, the xCELLigence software provides a method to analyze cell proliferation, adhesion, morphology, and death in real time. Changes in cell size, cell number, and cell adhesion result in changes of impedance, which are reflected as cell index (CI). Every condition was tested in triplicates, and the assay was performed three times.

#### TCGA-Bulk transcriptomics analysis

RNA-Bulk-seq data from 183 pancreatic cancer samples were downloaded from The Cancer Genome Atlas (TCGA-PAAD; phs000178) database. Raw unstranded count data were compiled into a matrix and clinical metadata were integrated for 178 of these patients. 5 patients of the 183 missed the Case-ID, which was needed to integrate the clinical metadata. All 183 samples were uploaded to the UniApp platform, where data preprocessing and normalization were conducted using the trimmed mean of M-values (TMM) method. Subtyping into basal and classical subtypes was performed using hierarchical clustering of a feature heatmap constructed from the Chijimatsu gene signature [48]. Euclidean distance and complete linkage were used for clustering, with auto-scaling applied to the gene expression values. Columns (samples) were grouped, and clusters were manually defined based on the dendrogram, resulting in 25 basal, 13 classical and 89 mixed samples (127 total) for Kaplan-Meier analysis (56 samples with low signature expression and 5 samples without Case-ID were excluded). Differential gene expression analysis (DGEA) and gene set enrichment analysis (GSEA) were performed to compare basal and classical subtypes. For survival analyses, Kaplan-Meier curves were generated to assess overall survival. Time-to-event was defined as time from diagnosis to death or last follow-up.

#### Metabolomics - mass spectrometry and metabolic profiling

Organoids were cultured at a density of 1 million cells in 14 Matrigel drops per well of a 6-well plate, with 2 mL organoid media per well. On day 5, organoids were treated with KAN0438757 at 25 µM for 24 h. Subjecting organoids to this concentration allowed us to capture acute/early primary metabolic drug effects. After 24 h of treatment, media was removed, and 4 mL of Cell Recovery Solution (CRS, Corning) was added to each well. Organoids were transferred to 15 mL Falcon tubes, pipetted 20 times, and incubated for 5 min with an additional 3 mL of CRS. After adding cold 0.9% NaCl, samples were centrifuged at 500 g for 3 min at 4 °C, supernatants were aspirated, and pellets were flash-frozen in liquid nitrogen, then stored at -80 °C. Metabolites were extracted on dry ice/ice with 800 µL cold 62.5% methanol in water and 500 µL ice cold chloroform. The samples were vortexed at 4 °C to extract metabolites, centrifuged for 10 min, and the phases were separated. For measurements of polar metabolites, the methanol water phase was dried at 4 °C by vacuum centrifugation and resuspended in water LC-MS grade, and analyzed in a Dionex UltiMate 3000 LC System (Thermo Scientific) with a thermal autosampler set at 4 °C, coupled to a Q Exactive Orbitrap mass spectrometer (Thermo Scientific). A volume of 10 µL of sample was injected on a C18 column (Acquity UPLC HSS T3 1.8 μm 2.1 × 100 mm). The separation of metabolites was achieved at 40 °C with a flow rate of 0.25 ml/min. A gradient was applied for 40 min (solvent A: 10mM Tributyl-Amine, 15mM acetic acid – solvent B: Methanol) to separate the targeted metabolites (0 min: 5% B, 2 min: 5% B, 7 min: 37% B, 14 min: 41% B, 26 min: 95% B, 30 min: 95% B, 31 min: 5% B, 40 min: 5% B. The MS operated in negative full scan mode (m/z range: 70–900) using a spray voltage of 4.8 kV, capillary temperature of 300 °C, sheath gas at 40.0, auxiliary gas at 10.0. Data was collected using the Xcalibur software (Thermo Scientific), followed by a custom MATLAB script analysis (version 2023a).

### PDX generation and in vivo treatment

For PDX experiments, 1–2 mm³ tumor fragments from two PDAC models, PDXGO12 (TM009; F7; ICD-10 C25.0; 64-year-old at diagnosis; R0; pT3 pN1 cM0 L0) and PDXGO4 (TM002; F4; ICD-10 C25.0; 80-year-old at diagnosis; R1; pT2 pN1 cM0 L1), were subcutaneously engrafted into the flanks of NRG mice (NOD.Rag1−/−; Il2rg−/−; JAX #007799) (F0). Tumor growth was measured three times per week with calipers and volume calculated as (length × width²)/2 (mm³). Primary outgrowths were harvested at ~ 1,500 mm³ and passaged as secondary xenografts (F1). For RNA extraction and RNA-sequencing analysis, outgrowths from untreated PDX models (TM009, F2, F4 and TM002, F1) were used. Randomization and treatment were initiated when implanted tumors reached ~ 100–200 mm³. Vehicle controls (*n* = 6) received intraperitoneal (i.p.) injections of 20% ethanol, 20% PEG-400, 20% Phosphal-50, 20% DMSO, and 20% 0.9% NaCl. The KAN0438757 group (*n* = 6) received 25 mg/kg KAN0438757 i.p. (10 mg/mL in 60% DMSO/40% 0.9% NaCl) on a 3-days-on/3-days-off schedule, as previously described in [49]. The gemcitabine group (*n* = 6) received 50 mg/kg gemcitabine i.p. (10 mg/mL in 0.9% NaCl) as a single dose every other week (see Fig. S7G). The submaximal dose of 50 mg/kg gemcitabine was chosen based on our previous experience using immunosuppressed mice and on animal studies using PDAC xenografts and metabolic inhibitors [49–51].

Animals were euthanized 24 h after the final injection treatment, and tumors were fixed in 4% paraformaldehyde for downstream analyses. All procedures were approved by the German *Niedersächsisches Landesamt für Verbraucherschutz und Lebensmittelsicherheit* (LAVES; TVA 23.00443 and TVA17.2407).

### Quantification and statistical analysis

In addition to bioinformatical approaches described above for spaRNA-seq and bulk RNA-seq, all other statistical analyses were performed using GraphPad Prism 8.2.1. For survival analyses, the Gehan-Breslow-Wilcoxon test was used to calculate p-values between groups, accounting for differences in early survival events, and the Kaplan-Meier method was used to plot survival curves. Results were considered statistically significant when p-values < 0.05 (*, *p* < 0.05; **, *p* < 0.01; ***, *p* < 0.001; ****, *p* < 0.0001). Multiple comparisons were not applied.

Correlation analysis for the multiplex staining results was based on Spearman’s rank correlation test (Spearman’s *ρ)*. Moderate correlation was assumed at *ρ > 0.3* and strong correlation at *ρ > 0.5.*

Protein expression levels were evaluated for statistical significance using Kruskal–Wallis tests, followed by Dunn’s post-hoc multiple-comparison correction for each marker. Subsequently, p-values were adjusted to control the false discovery rate (FDR) at a threshold of p_adj_<0.05 using the two-stage step-up procedure proposed in [52].

### Graphics created using external-party features

**Fig. 1:**
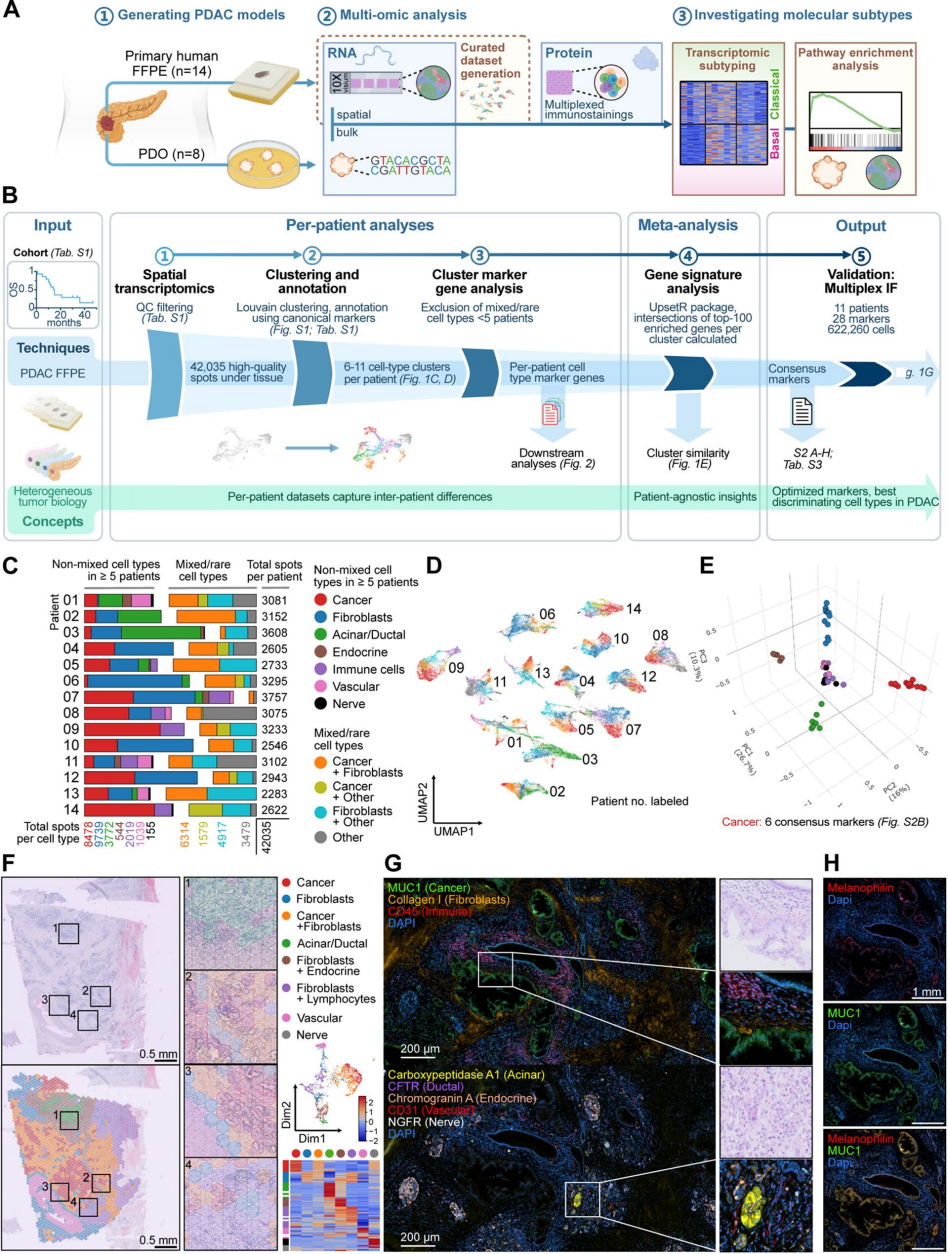
Spatial transcriptomics analysis reveals robust marker genes for common PDAC cell types. (**A**) Overview of the experimental workflow for dataset generation, and subtype investigation, summarizing the key models used (1-2) and aims of the study (3). (**B**) Workflow illustration for patient-wise cell type annotation and intersection-based meta-analysis. (**C**) Patient-wise composition of spot identities and total spot numbers per patient and cell type. (**D**) UMAP plot of all 42,035 spots across all 14 patients, annotated by cell type (including all mixed and pure cell types), colored according to legend in Fig. 1C. (**E**) Three-dimensional principal component analysis (PCA) on the pairwise Jaccard similarity coefficients of the top 100 enriched marker genes for cancer, fibroblast, endocrine, acinar/ductal, nerve, immune cell, and vascular cell clusters. (**F**) H&E staining, spatial cluster overlay, and UMAP plot of cell types in exemplary patient 13, alongside a heatmap of canonical cell type marker gene expression in clusters of patient 13 (Table S2). The combined heatmap for all patients is shown in Fig. S2B. (**G**) Multiplexed MACSima immunostaining of patient 07, validating cell type annotations. (**H**) Immunostaining for the novel PDAC marker Melanophilin (MLPH) and established PDAC marker MUC1 in exemplary patient 07

Figures were created using graphical assets from BioRender. Graphical abstract and Fig. 1: Gätje, F. (2025) https://BioRender.com/z8dpmf4; Fig. S7G: De Oliveira, T. (2025) https://BioRender.com/f6zrc5a.

#### Lead contact

Further information and requests for resources and reagents should be directed to and will be fulfilled by the lead contacts, Lena-Christin Conradi (lena.conradi@med.uni-goettingen.de) and Tiago De Oliveira (tiago.deoliveira@med.uni-goettingen.de).

### Experimental model and study participant details

#### Patient samples

For generation of FFPE tissues and PDOs, surgically resected PDAC tissue was derived from patients between 2020 and 2022 (Table S1). Samples were collected at the University Medical Center Göttingen (UMG) under ethical approval of the UMG (ethical vote number 25/3/17).

## Results

### Spatial transcriptomics analysis reveals robust marker genes for common PDAC cell types

We assessed the molecular features of PDAC’s different cellular components at a spatial resolution, with a particular focus on metabolism. Therefore, we conducted spaRNA-seq on formalin-fixed paraffin-embedded (FFPE) tissues from 14 PDAC patients who had undergone surgical resection without pretreatment (Table S1). This generated a comprehensive dataset validated by multiplexed immunofluorescence staining for well-established and newly identified marker genes on the same resected samples. Additionally, a cohort of PDOs was established to validate cancer cell-related findings via bulk RNA sequencing (Fig. 1A). The tissue slides chosen for spaRNA-seq contained histologically identifiable components, including cancer cells, desmoplastic stroma, and normal pancreatic parenchyma. Our dataset comprises 42,035 tissue-covered spots that passed quality control (Table S1), the workflow for annotation of clusters and intersection-based meta-analysis of identified cell-types is illustrated in Fig. 1B.

First, we independently annotated the spots per specimen by applying Louvain clustering to uniform manifold approximation (UMAP)-reduced data and examined canonical marker expression for each cluster (Figs. S1A and S2A-B, Table S2). Our analysis revealed varying cell type proportions per sample, including mixed clusters at tissue boundaries, such as clusters containing both cancer cells and fibroblasts, reflecting the infiltrative nature of PDAC, with typically disseminated growth and a strong desmoplastic reaction (Figs. S1A and S2A-B). Six to eleven cell type clusters were identified per patient. We identified 7 pure (non-mixed) cell types present in at least five patients and selected these for further evaluation (Fig. 1C). For subsequent analyses, we merged the entire dataset to derive more generalizable results that were consistent across the cohort. Dimension reduction of all spots resulted in sample-specific clustering, capturing individual gene expression profiles for each PDAC patient (Fig. 1D).

Jaccard similarity principal component analysis of the top 100 enriched marker gene sets for each cell-type cluster within individual patients visualized distinct arrangements for cancer areas, fibroblastic stroma, acinar/ductal parenchyma, and pancreatic islet endocrine cells across different samples, with the strongest discrimination visible between cancer cells and fibroblasts (Fig. 1E). By contrast, immune, vascular, and nerve clusters appeared to lie closer to one another and exhibited individual affinities to cancer, fibroblasts, and acinar/ductal clusters, consistent with their embedding within these tissues (Fig. 1E).

Prioritizing shared, highly enriched genes per cell type across patients, which could serve as robust cell type markers, we focused on the computed intersection sizes per cell type in the meta-analysis visualized by UpSet plots (Figs. S3A–H). Especially cell types present in normal pancreatic tissue, like endocrine cells, Schwann cells in nerve clusters, acinar/ductal cells and vascular cells showed larger intersection sizes, reinforcing inter-patient similarity of these cell types (Figs. S3A-H). The cancer cell clusters displayed higher inter-patient heterogeneity, with six common marker genes identified among all 14 samples (Fig. S3B and Table S3). Among these markers, four (*TSPAN8* [53], *KRT8* [54], *MUC1* [55], *FXYD3* [56]) are known markers for poor prognosis in PDAC, while *LGALS4* [57] predicts positive prognosis.

The specificity of MUC1 as a PDAC cancer marker is notable, given its use in clinical trials for dendritic cell vaccination to enhance immune response against cancer cells [58]. Furthermore, several preclinical trials employ MUC1 as a cancer cell marker in targeted PDAC treatments like IgE-based therapeutics [59] or radioimmunoconjugates [60]. Interestingly, Melanophilin (MLPH), classically required for melanosome transport [61] and previously associated with epithelial-to-mesenchymal transition (EMT), cell motility and invasion in various cancers [62, 63], emerged as a defining marker in our tumor cell cluster (Figs. S3B and S3I). In addition, to confirm tissue assignments by histology (Fig. 1F), we performed multiplexed immunofluorescence stainings (Fig. 1G). Remarkably, MLPH and MUC1 exhibited nearly identical, tightly overlapping expression patterns in malignant epithelial cells (Fig. 1H), with rare MLPH-positive stromal cells being identified, likely reflecting baseline expression, confirming its cell-type specificity. Using GRNboost2-based gene regulatory network analysis within cancer clusters, we identified a set of genes whose expression was positively associated with MLPH and that have been previously linked to tumorigenesis, metastasis, and poor prognosis in PDAC. These included genes involved in metabolic reprogramming (*PGC*,* PFKFB3*) or cell adhesion and migration (*GOLM1*,* ANXA10*). Given its rate-limiting role in glycolysis, *PFKFB3* emerged as a druggable candidate in PDAC (Fig. S3I). Conversely, genes involved in extracellular matrix (ECM) remodeling and tumor-stroma interactions, particularly collagens (*COL3A1*,* COL1A2*,* COL10A1*), showed negative expression-based associations with MLPH (Fig. S3I). Additionally, correlation analysis including all cells confirmed a strong association between MLPH and MUC1 expression (Spearman’s ρ = 0.80, *p* < 0.001). In comparison, the correlation between MUC1 and CD66c (CEACAM6), another well-established PDAC biomarker [64, 65], was lower (ρ = 0.36, Fig. S3J). Notably, MLPH exhibited stronger correlation with cancer markers than observed between established cancer markers, while maintaining lower correlations with other cell type markers, supporting its specificity (Fig. S3J).

Overall, our cross‑patient meta‑analysis delineates a set of highly specific marker genes for major PDAC cell types, refining insights into the tumor’s molecular landscape and establishing MLPH as a robust spatial marker of PDAC tumor cells.

### Cell type characterization unveils metabolically distinct cancer cells and functionally diverse subtypes of CAFs

**Fig. 2:**
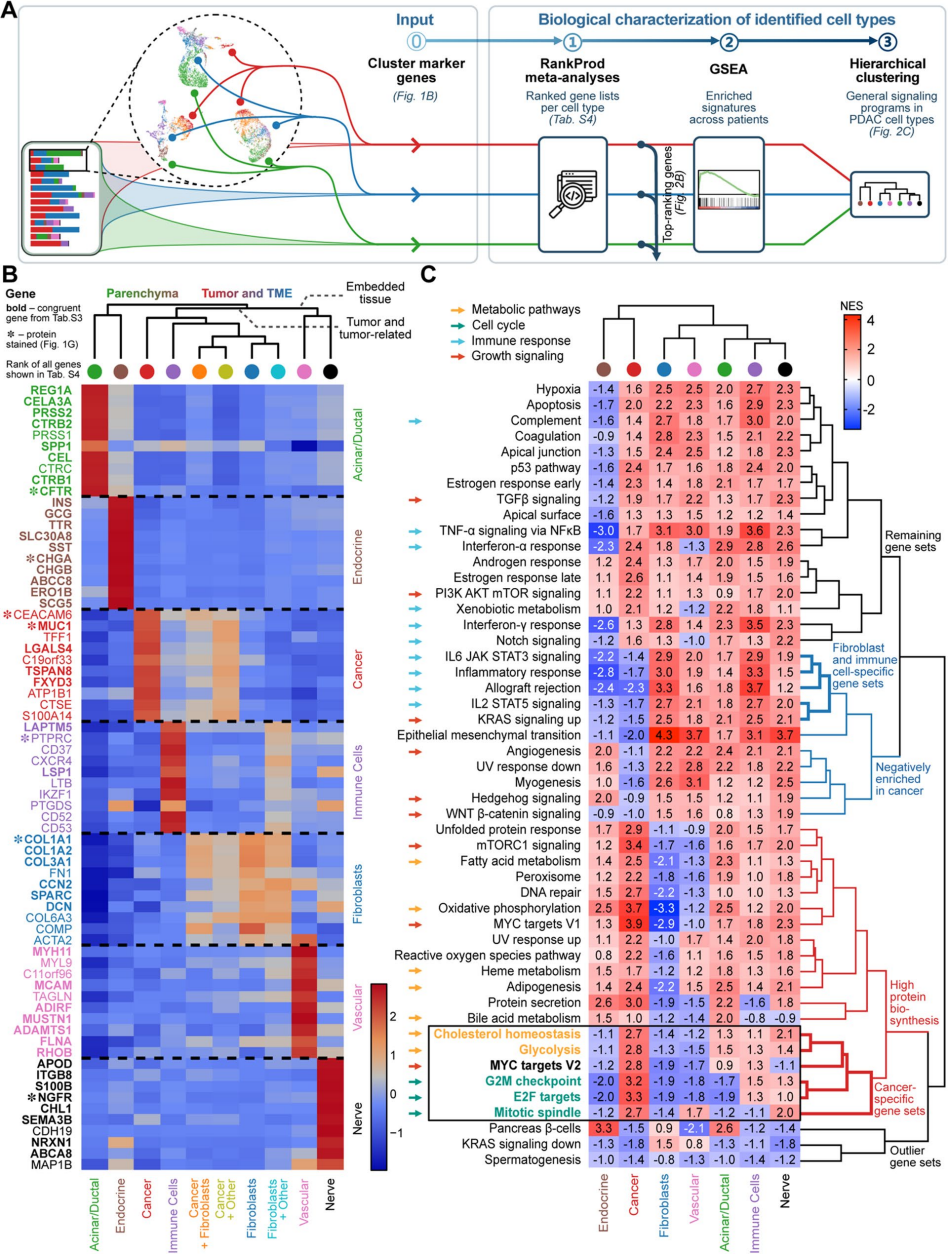
Cell type characterization unveils metabolically distinct cancer cells and functionally diverse subtypes of CAFs. (**A**) Workflow illustration for rank-based cell type characterizations. (**B**) Heatmap showing expression of the top 10 marker genes from each RankProd meta-analysis, performed for the 7 previously identified cell type clusters. Expression is shown across 10 cell types and cell type combinations in all 14 patients. Genes, for which the corresponding protein is stained via multiplexed MACSima immunostaining, highlighted by an asterisk (congruent marker genes, within the top 100 enriched genes in all patients, highlighted in bold). Dendrogram branches highlight clusters identified by multiple bootstrapping. Color scale: red, high expression; blue, low expression. (**C**) Heatmap showing enrichment of MSigDB Hallmark pathways in the 7 cell types based on RankProd meta-analyses. Normalized enrichment score (NES) is annotated. Gene sets in chosen categories of cellular signaling annotated by colored arrows. Colored dendrogram branches highlight clusters identified by multiple bootstrapping. Color scale: red, high NES; blue, low NES

Having confidently annotated tissue types within the dataset, we sought to characterize the cell types by analyzing their global transcriptional profiles for underlying biological processes (Fig. 2A). To that end, we performed RankProd meta-analyses per cell type, ranking enriched genes in the respective clusters across patients. This yielded marker gene lists for common cell types in PDAC, ranked by their expression levels across patients (Table S4).

To demonstrate cell type specificity, we visualized the expression of the top 10 ranked genes per cell type (Fig. 2B). Notably, MLPH ranked 15th in the cancer cell clusters (Table S4). Hierarchical clustering of these expression profiles further distinguished pancreatic parenchymal cell types (acinar/ductal, endocrine) from tumor and microenvironmental compartments, including cancer cells, immune cells, fibroblasts, vascular clusters, nerve clusters, and mixed clusters (Fig. 2B).

Next, we assessed the biological processes in pure, non-mixed cell type clusters by performing gene set enrichment analysis (GSEA) on the ranked gene lists per cell type. Relationships between cell types and hallmark pathways were visualized by hierarchical clustering (Fig. 2C). Interestingly, several hallmark gene sets [64] were enriched in cancer cell clusters, including cell cycle programs (E2F targets, G2M checkpoint, mitotic spindle), Myc-targets, and two distinct metabolic pathways (cholesterol homeostasis, glycolysis). These findings underscore the highly proliferative nature of PDAC cancer cells, where cholesterol homeostasis supports membrane synthesis, and glycolysis meets elevated energy demands required for rapid cell division [66] (Fig. 2C). Furthermore, our analysis revealed two distinct metabolic profiles in cancer cell clusters: (i) glucose metabolism (glycolysis) and (ii) lipid metabolism (fatty acid metabolism, adipogenesis, cholesterol homeostasis). Thereby, cholesterol metabolism showed a more exclusive expression pattern for cancer cells, while fatty acid metabolism and adipogenesis were additionally enriched in other cell-types, such as acinar/ductal cells or endocrine cells (Fig. 2C, yellow highlights).

Given that the desmoplastic reaction is a hallmark of PDAC pathogenesis [67], we then focused on the fibroblast-specific transcriptional signatures (Fig. 2B and Table S4). Our analysis shows markers upregulated in fibroblasts, which actively contribute to tumor biology, beyond connective functions. Notably, *FN1* (fibronectin) emerged as a central network hub in this gene set (Fig. 2B and Table S4). This major ECM protein appears to orchestrate fibroblastic programs – including SPARC and DCN – and has been linked to aggressive PDAC features [68]. SPARC, predominantly localized within the tumor stroma, is associated with poor prognosis and displays a complex, context-dependent role in PDAC, exhibiting both oncogenic and tumor-suppressive properties in experimental models. Nevertheless, elevated stromal SPARC secretion is consistently correlated with adverse outcomes across multiple tumor types, underscoring its potential as a marker of aggressive disease biology [69, 70].

Additionally, collagen genes were prominently overexpressed: *COL1A2* (with *COL1A1*) was highly expressed in the desmoplastic stroma, reinforcing ECM integrity, whereas *CCN2* (CTGF) and *ACTA2* (α-SMA) were co-expressed, indicating an activated myofibroblastic CAF (myCAF) phenotype (Fig. 2B, Table S4). These myCAF-associated markers (high α-SMA expression, collagen-rich) contrast with inflammatory CAFs (iCAFs), which lack α-SMA but secrete cytokines such as IL6 and IL11. Consistently, hierarchical clustering of gene sets revealed enrichment of IL6–JAK–STAT3 signaling and other inflammatory pathways (e.g., IL2-STAT5, IFN-gamma response), suggesting that the identified clusters comprise a mixture of myCAF and iCAF subtypes, each contributing to PDAC’s complex microenvironment (Fig. 2C and Table S4).

To delineate the molecular communication between fibroblast clusters and cancer cells, we applied ligand-receptor interaction analysis using CellPhoneDB (Fig. S3K). Fibroblast clusters prominently engaged with tumor cell clusters via growth factor signaling axes such as *HGF*–*CD44*, *FGF*-mediated signaling, and *VEGF*-mediated signaling, all of which are known to drive proliferation, angiogenesis, and EMT in PDAC [71]. Additionally, we identified a robust *SPP1*–*CD44* interaction, a known CAF-mediated mechanism promoting cancer cell stemness and late-stage aggressiveness [72]. Beyond these pro-tumorigenic signals, fibroblast clusters also contributed to immune evasion via *MIF* and *LGALS9*, interacting with *TNF* receptors and *CD44* on cancer cells [73, 74].

Collectively, our spatial transcriptomic and ligand-receptor analyses define PDAC by highly proliferative, metabolically reprogrammed cancer cells that co-occupy niches with functionally diverse CAF subtypes, being myCAFs governed by an *FN1*-*SPARC*-*DCN* ECM hub and iCAFs dominated by IL6-JAK-STAT3 signaling. Through growth-factor (*HGF*, *FGF*, *VEGF*) and immunoregulatory (*SPP1*–*CD44*, *LGALS9*–*CD44*) axes, these stromal populations orchestrate an immunosuppressive, therapy-resistant niche characteristic of advanced disease. Our dataset captures the complexity of PDAC tumor–stroma crosstalk and nominates ECM components and associated signaling pathways as tractable biomarkers and therapeutic targets.

### Patient stratification into basal and classical PDAC subtypes with distinct pathway activities

**Fig. 3:**
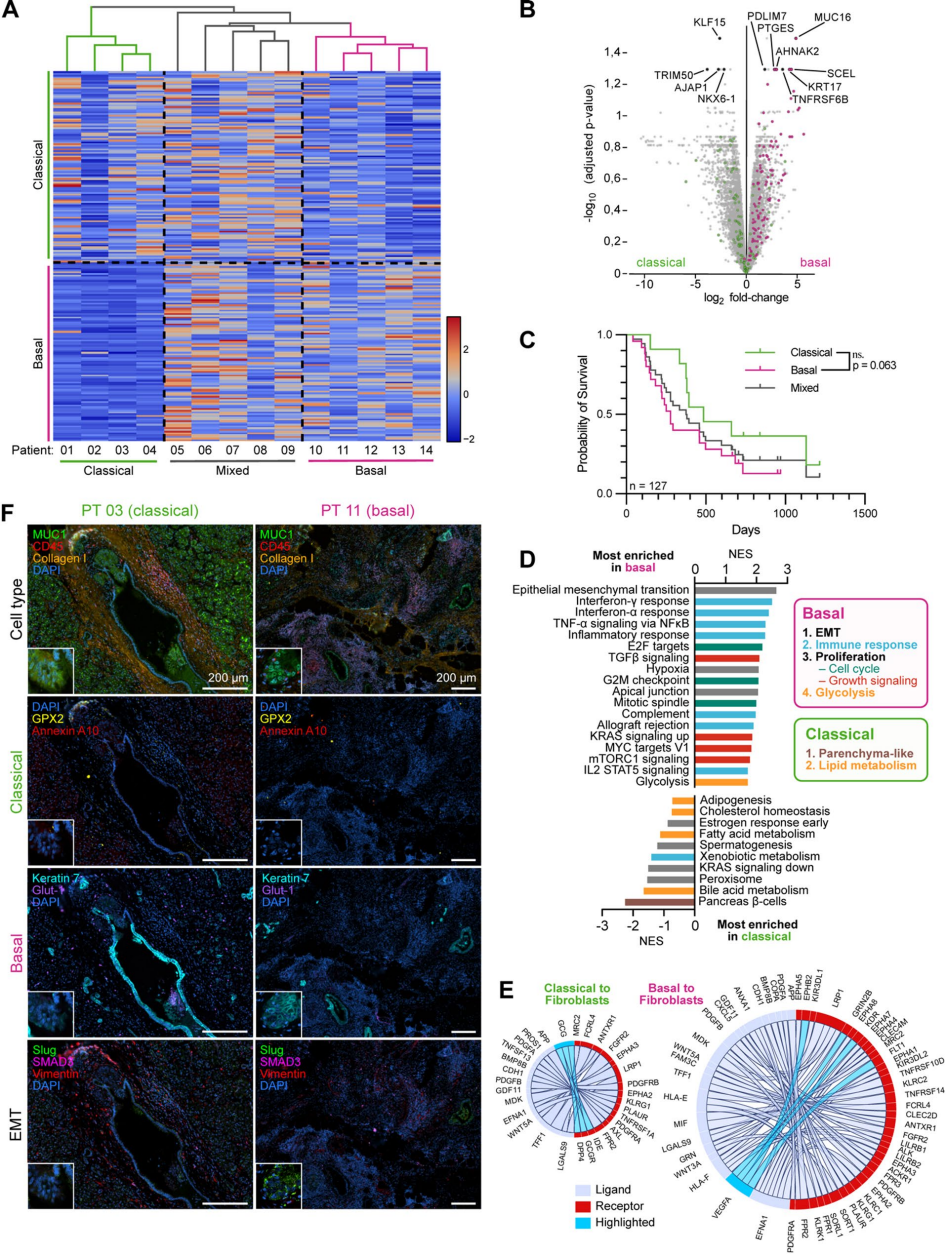
Patient stratification into basal and classical PDAC subtypes with distinct pathway activities. (**A**) Heatmap showing hierarchical clustering of cancer cell clusters from 14 PDAC patients according to established basal/classical gene signatures, stratifying patients into classical, basal, and mixed subtypes. Colored dendrogram branches indicate clusters identified by multiple bootstrapping. Color scale: red, high expression; blue, low expression. (**B**) Volcano plot showing differentially expressed genes of cancer cell clusters in basal versus classical patients, following pseudobulk adjustment of the spatial transcriptomics dataset. Selected genes labeled and highlighted in black, genes in the established basal and classical signatures highlighted in pink and green, respectively. (**C**) Survival analysis of PDAC subtypes in the TCGA-PAAD cohort. Molecular subtyping of TCGA-PAAD was performed analogously to spatial PDAC patients (see Fig. S4A). n=127 (25 basal, 13 classical, 89 mixed). (**D**) Waterfall plot: Most enriched MSigDB Hallmark gene sets in cancer cell clusters of basal versus classical patients (patient-level analysis). Pathways are grouped by functional categories, indicated by colored bars. (**E**) Cell-cell interaction between cancer cells and fibroblasts in classical and basal subtyped patients, using CellPhoneDB. (**F**) Protein expression of subtype markers and EMT markers in primary tumor tissue of exemplary patients using multiplexed MACSima immunostaining

To explore inter-patient heterogeneity within the cancer cell clusters, we first stratified patients into basal and classical PDAC subtypes based on recently established single-cell refined gene signatures derived from six independent datasets (~ 130,000 cells) [48]. Hierarchical clustering of spatially-resolved cancer cell populations identified five basal, four classical, and five mixed subtype patients (Fig. 3A).

Basal cancer cell clusters upregulated transcripts linked to tumor promotion and poor prognosis in PDAC, including *KRT17* [75], *AHNAK2* [76], *SCEL* [77], and *MUC16* [78] (Fig. 3B, Table S5). Prior studies also implicate these genes in growth-factor signaling (*PLAG1* [79]), proliferation and migration (*KRT17* [80], *SCEL* [77], *MUC16* [78]), EMT, and metabolic rewiring toward glycolysis (*PTGES* [81, 82]). We also observed strong induction of *PDLIM7* and *TNFRSF6B*, which drive proliferation, migration, and invasion in other malignancies, although their pro-tumorigenic roles in PDAC remain to be defined [83, 84] (Fig. 3B and Table S5). Conversely, classical clusters were enriched for transcripts associated with less aggressive biology and favorable prognosis, including *TRIM50* [85], *AJAP1* [86], and *KLF15* [87]. Notably, *TRIM50* limits proliferation, motility, and EMT in PDAC [85], *KLF15* suppresses stemness [87], and *AJAP1*/*NKX6-1* have tumor-suppressive/positive-prognostic associations in other cancers [86, 88, 89] (Fig. 3B and Table S5).

To externally cross-validate our gene signature, we have analogously performed the corresponding molecular subtyping in the TCGA-PAAD cohort phs000178 [90] (Fig. S4A). Even though not reaching significance, survival analysis according to this gene signature showed a trend towards worse prognosis in the basal subtype, aligning with previous reports (Fig. 3C).

Corroborating these findings, GSEA in our spatial transcriptomics cohort showed basal clusters enriched in pathways linked to poor prognosis, including EMT and proliferation (E2F-targets, G2M checkpoint, mitotic spindle), TGF-beta signaling, KRAS signaling up, Myc targets, and mTORC1 signaling (Fig. 3D). Additionally, basal patients upregulated immune-responsive pathways (interferon-γ and -α response, TNF-α signaling via NFкB, complement) and glycolysis. Conversely, classical cancer cell clusters upregulated pathways linked to normal pancreatic tissue (pancreas β-cells) and lipid metabolism (adipogenesis, cholesterol homeostasis, fatty acid metabolism, bile acid metabolism) (Fig. 3D).

Next, we assessed subtype-specific communication of cancer cell clusters with fibroblast clusters using CellPhoneDB, revealing distinct interaction patterns between basal and classical clusters with fibroblasts and autocrine signaling (Fig. 3E).

Interestingly, classical cancer cell clusters showed endocrine-like signaling toward fibroblasts in an autocrine manner, which was absent in basal clusters (Figs. 3E and S4B). Specifically, classical clusters expressed insulin (*INS*) and glucagon (*GCG*) along with their receptors *INSR* and *GCGR*, suggesting autocrine/paracrine signaling loops. Previous findings have identified *INS/GCG* double-positive cancer-associated endocrine cells as mediators of tumor-initiation and tumor growth rather than drivers of invasion and metastasis [91]. In line, the higher *INS/INSR* signaling in the cancer cell compartment of the classical subtype is consistent with an anabolic state. This may arise from partial islet-lineage differentiation or from endocrine-like cell infiltration providing paracrine insulin, thereby promoting tumor growth. Conversely, basal cancer cell clusters preferentially activated pro-angiogenic signaling toward fibroblasts: *VEGFA* was a dominant ligand in basal cancer-to-fibroblast interactions, whereas classical tumors showed minimal *VEGF* activity (Fig. 3E). This pattern aligns with the hypoxic, glycolytic, highly proliferative basal phenotype [92].

To validate our findings in vitro, we established a cohort of 8 PDO lines, including 3 from primary tumor specimens included in our spatial transcriptomics cohort (Table S1). Bulk RNA sequencing followed by the same signature-based hierarchical-clustering approach used for the spatial transcriptomics identified 3 basal, 3 classical, and 2 mixed subtype PDOs. This finding was corroborated by GSEA comparing basal and classical PDOs (Fig. S4C). Basal PDOs were enriched for immune pathways (interferon‑γ and ‑α responses, TNF‑α signaling via NF‑κB, inflammatory response, complement activation) and TGF‑β signaling (Fig. S4D). In contrast, classical PDOs exhibited signatures characteristic of normal pancreatic parenchyma, notably β‑cell signaling. Unexpectedly, they also showed heightened EMT, activation of proliferative pathways (G2/M checkpoint and mitotic spindle), and KRAS signaling, diverging from patterns observed in primary tumor tissues (Fig.. S4D). This apparent compensatory behavior may reflect the absence of key microenvironmental components such as fibroblasts, immune cells, and endothelial cells that normally contribute to EMT and shape the tumor ecosystem. When leveraging the larger subtyped TCGA-PAAD cohort, whose whole-tumor RNA-sequencing incorporates cancer and stromal compartments due to its bulk sequencing nature, our spatial cancer cell signals were reproduced: GSEA contrasting basal versus classical patients showed enrichment of EMT, immune pathways, and hypoxia in basal cases, whereas lipid-metabolism pathways predominated in classical cases (Fig. S4E).

Finally, multiplexed immunostainings visualized protein-level expression patterns of subtype and EMT markers in tumor tissues (Fig. 3F). MUC1, COL1, and CD45, which occurred among the top-ranking cell type markers across all patients, enabled reliable cell type allocation (see also Figs. 1G and 2B). The classical markers GPX2 and AnnexinA10, previously identified in spatial transcriptomics and members of the established single-cell refined subtype gene signatures [48], showed higher expression on protein level in classical patients (Table S5). Unexpectedly, the basal markers Keratin-7 and Glut-1 [48] showed focal enrichment in both subtypes, with Keratin-7 being mainly expressed in cancer cells. EMT markers Slug, SMAD3, and VIM were found focally enriched in stromal areas of both subtypes (Fig. 3F).

In summary, our transcriptional profiling stratifies PDAC cancer cells into basal and classical subtypes in both primary tissue and PDO models. Basal cells are characterized by high proliferation, invasion, and immune response, whereas classical cells resemble normal pancreatic parenchyma, exhibiting endocrine signaling and lipid metabolism.

### Intra-tumoral heterogeneity and subtype plasticity of PDAC cancer cells

**Fig. 4:**
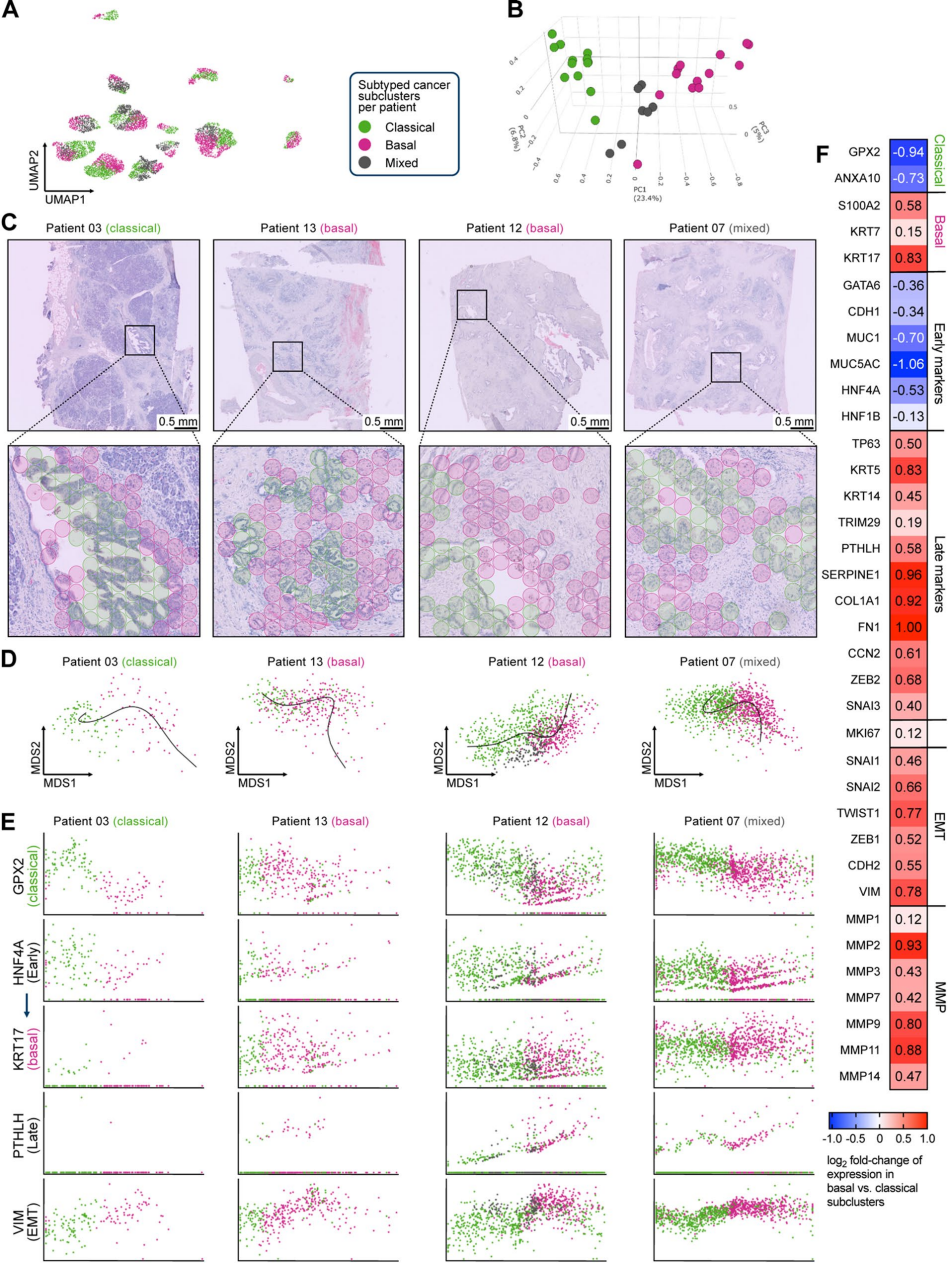
Intra-tumoral heterogeneity and subtype plasticity of PDAC cancer cells. (**A**) UMAP visualization of cancer cell clusters from all patients (except patient 02), displaying the composition of classical, basal, and mixed cancer subclusters. Subclustering and subtyping of cancer subclusters were performed analogously to whole patient clustering and subtyping (see also Fig. 3A). Subclustering for exemplary patient 07 is shown in Figure S5A. (**B**) Jaccard similarity analysis based on the top 100 enriched marker genes of classical and basal cancer subclusters, performed analogously to the cell type similarity analysis (see also Figs. 1B and 1E). (**C**) Overlay of cancer cell subclusters on H&E images of exemplary patients, showing spatial distribution of classical, basal, and mixed cancer regions within the tumors. (**D**) Pseudotime trajectory analysis of cancer subclusters in the same patients from (**C**), inferred with SCORPIUS using all genes and visualized in the first two multidimensional scaling (MDS) dimensions based on Spearman distances. (**E**) Expression dynamics of selected subtype markers, early and late PDAC progression and EMT, marker genes projected along the inferred pseudotime trajectories. (**F**) Heatmap showing pseudobulk-adjusted differential expression of various marker genes in basal versus classical cancer cell subclusters, presented as log2 fold-change. Color scale: red, high expression; blue, low expression

Exploring intra-tumoral heterogeneity (ITH) in PDAC via transcriptomic profiling is critical for revealing distinct cellular subpopulations, providing mechanistic insights into niche-specific programs related to treatment response and failure [93]. Recently published datasets utilizing spatially-resolved transcriptomics have already uncovered relevant findings on PDAC aggressive biology and plasticity [27–29], however, key aspects of ITH warrant further elucidation. Therefore, to assess ITH within the spatial cancer cell clusters, we performed sub-clustering and subtype annotation across all cancer spots per patient, identifying classical, basal, and mixed cancer subclusters in 13 out of 14 patients (all except PT02) (Fig. 4A). Subtyping of cancer subclusters was performed as previously described. Exemplary subclustering for patient 07 is shown in Figure S5A and most enriched hallmark gene sets in basal versus classical cancer subclusters across patients are shown in Figure S5B, refining the general trends of differentially expressed pathways that were already observed in patient-level analyses.

To assess the transcriptional associations between these subclusters, we performed Jaccard similarity analysis using the top 100 enriched marker genes per classical/basal subcluster, mirroring the approach in Fig. 1E. This revealed minimal overlap between classical and basal subtypes, underscoring their distinct transcriptional programs (Fig. 4B). Spatial mapping revealed classical, basal, and mixed regions, with basal‑enriched spots localizing at invasive fronts and poorly differentiated areas (Fig. 4C), denoting a spatial link between basal identity and tumor aggressiveness.

Previously, integrated whole-genomic-based studies (WGS) reported that, at single-cell resolution, most tumors contain both basal- and classical-like tumor cells [15]. However, spatial transcriptomic studies using gene signature-based subtyping are missing. Moreover, the concept that the classical signature represents a default path for pathogenesis and that the basal phenotype is the result of an evolutionary progression requires further exploration [15]. Therefore, to reconstruct how malignant populations evolve in situ, we sought an unbiased trajectory-inference method compatible with spot‑level transcriptomes. Applying the SCORPIUS algorithm [33] to all cancer subclusters per patient, we ordered all cancer spots along a linear pseudotime trajectory based on their transcriptomic signatures. Across patients, the trajectories displayed a reproducible pattern: spots with a classical expression program clustered at early pseudotime, basal spots at late pseudotime, and a continuum of mixed‑signature spots filled the intermediate space (Fig. 4D). This organization implies that the two canonical PDAC subtypes are not fixed compartments but rather endpoints of a transcriptional continuum, with intermediate transitional states.

Evaluation of kinetic markers supported this interpretation. The classical marker *GPX2* peaked at pseudotime origin and declined monotonically, while the basal marker *KRT17* rose reciprocally (Fig. 4E). Consistently, the early PDAC progression marker *HNF4A* peaked early — where classical spots predominate — and then declined, while the late‑stage marker *PTHLH* steadily rose toward the basal end of the trajectory (Fig. 4E). In parallel, *VIM*, *SNAI2*, *MKI6*7, and matrix metalloproteinases 2 and 9 (*MMP2/9*) increased along pseudotime (Figs. 4E and S5C). Differential gene expression analysis (DGEA) comparing all classical versus basal cancer subclusters confirmed higher early progression markers in the classical subtype and upregulation of late progression, EMT, proliferation, and MMPs in the basal subtype (Figs. 4F and S5C).

In summary, reciprocal, gradual trajectory-aligned shifts are more consistent with evolutionary progression than with spatial admixture, supporting the concept of intrinsic subtype plasticity whereby classical cancer cells acquire basal features as disease progresses.

### Highly active metabolic tumor niches define aggressive regions across PDAC subtypes

**Fig. 5:**
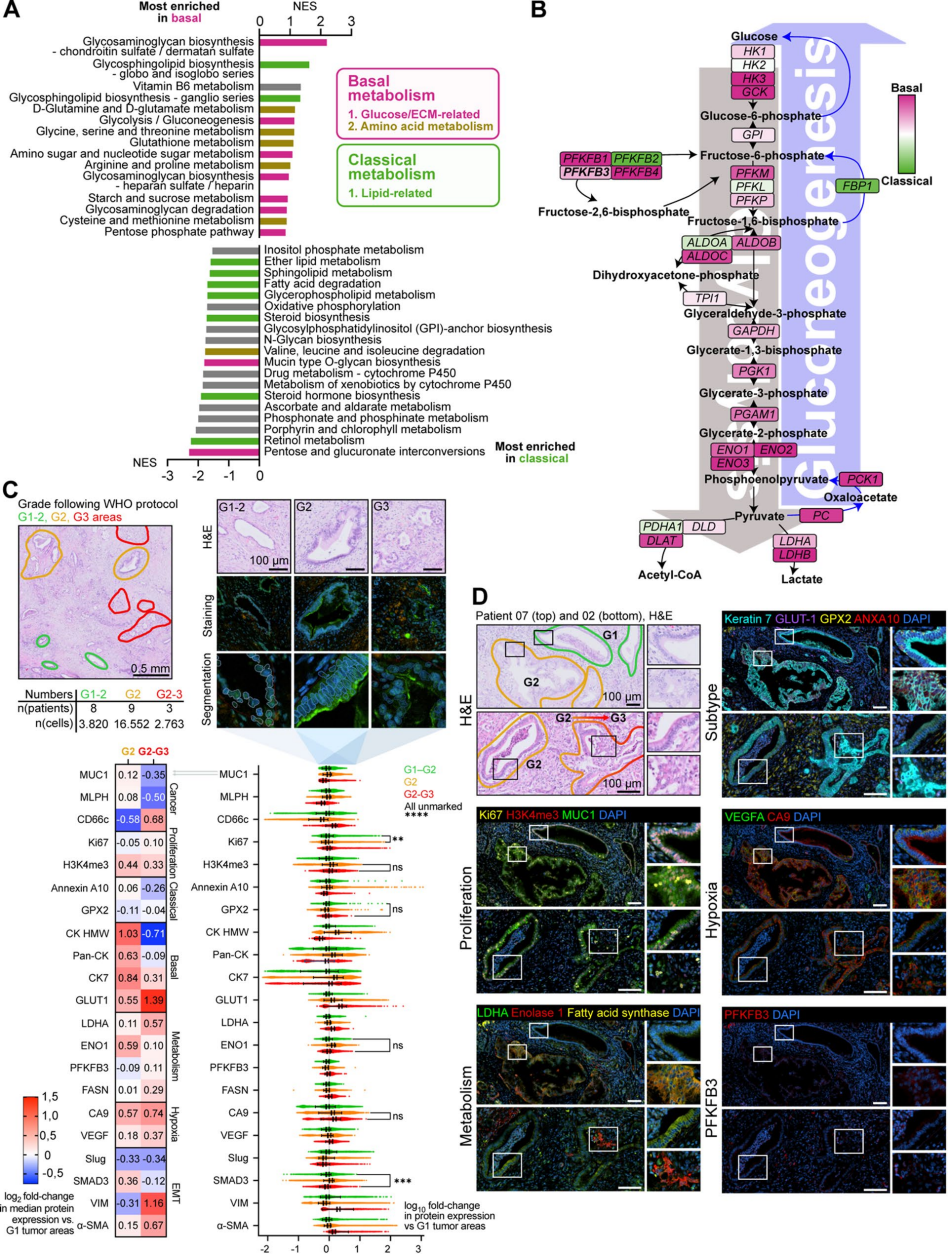
Highly active metabolic tumor niches define aggressive regions across PDAC subtypes. (**A**) Waterfall plot: Most enriched KEGG metabolism gene sets in basal versus classical cancer subclusters across all patients. Pathways are grouped by functional categories, indicated by colored bars. Comparison between the whole patient subtypes is shown in Figure S5E. (**B**) Glycolysis pathway mapping: differentially expressed genes between basal and classical cancer subclusters, mapped according to the glycolysis/gluconeogenesis pathway, color-coded according to scaled log fold-change values. Color scale: pink, basal; green, classical. Analysis for full patient subtypes is shown in Figure S5D. (**C**) Quantification of protein expression in high- to low-grade lesions across the dataset. Quantification was performed after cell segmentation within manually annotated regions based on histological morphology. Scatter plots compare fluorescence intensities, normalized per marker to the median intensity in the G1 cells. Data are presented as Median ± interquartile range. Kruskal-Wallis test with Dunn’s post-hoc multiple-comparison correction for each marker. P-values were further adjusted to control the false-discovery rate (padj<0.05) with the two-stage step-up procedure of Benjamini, Krieger and Yekutieli. Log2 fold-changes in median fluorescence intensity are shown by heatmap. (**D**) Immunostainings of selected markers in representative areas of high- and low-grade areas in exemplary PDAC patient tissues (patients 07 and 02)

Given the relevance of our previous findings on ITH and subtype plasticity, we decided to further evaluate whether molecular subtyping trajectories would also be accompanied by tumor histological progression. Thus, to spatially identify concurrence between acquisition of basal signature expression and basal-linked programs such as glycolysis (Figs. 5A-B and S5D-F) and EMT (Figs. 3D-E and S6A), with higher histological grade, we employed a complementary bottom-up approach, starting from unbiased histological grading, followed by quantitative multiplex immunostaining, in a total of 11 samples (Figs. 5C-D and S6B-C). Upon cell segmentation, fibroblast and tumor markers were thresholded to separate tumor from stroma for region-specific quantification. Notably, even though glycolysis is associated with the basal subtype and lipid anabolism with the classical PDAC subtype [94], subsequent multiplex staining showed that, independent of patient-level molecular subtype, high-grade lesions with basal-like features emerged as highly proliferative and metabolically “hot” niches, with significantly higher activity of both glycolysis and lipogenesis as well as increased hypoxia (Figs. 5C-D and S6C). H3K4me3 expression was markedly elevated in higher-grade lesions, consistent with increased transcriptional activity and possibly dysregulation of gene expression programs (Fig. 5C, heatmap and horizontal violin plots). Supporting dysregulation and dedifferentiation at higher grade, epithelial markers (MLPH, MUC1, CK HMW, Pan-CK, CK7) decreased, while mesenchymal markers (VIM, α-SMA) increased (Fig. 5C, all markers *p* < 0.0001, unless stated otherwise). Strikingly, the cancer cell marker CD66c showed upregulation in higher-grade areas. Co-staining of MLPH or MUC1 with CD66c revealed a grade-dependent shift from MLPH or MUC1 expression in lower-grade regions to CD66c expression in higher-grade areas, highlighting the need for combinatorial marker strategies to capture tumor heterogeneity (Fig. S6D).

Taken together, we identified aggressive niches characterized by elevated metabolism, proliferation, and hypoxia, suggesting increased angiogenesis. Notably, these niches are not exclusive to a subtype but were present across all PDAC patients, independent of their subtype.

### Targeting aggressive metabolic niches in PDAC through glycolysis inhibition

Given the identification of highly active metabolic tumor niches across both classical and basal PDAC subtypes, we next aimed to evaluate whether glycolysis inhibition could effectively target these aggressive regions independent of subtype. To this end, we employed KAN0438757, a selective inhibitor of PFKFB3 [95], a key glycolytic regulator that is known to be overexpressed in various cancers including PDAC [96] and found highly expressed in metabolically active cancer cell subclusters and high-grade tumor regions. Moreover, *S100A2* and *VEGFA*, both markers that are characteristic for high-grade lesions and highly proliferative, hypoxic environments, showed positive expression-based association with PFKFB3 in cancer cell clusters, observed in gene regulatory network analysis (Figs. 5C-D and S6E).

**Fig. 6:**
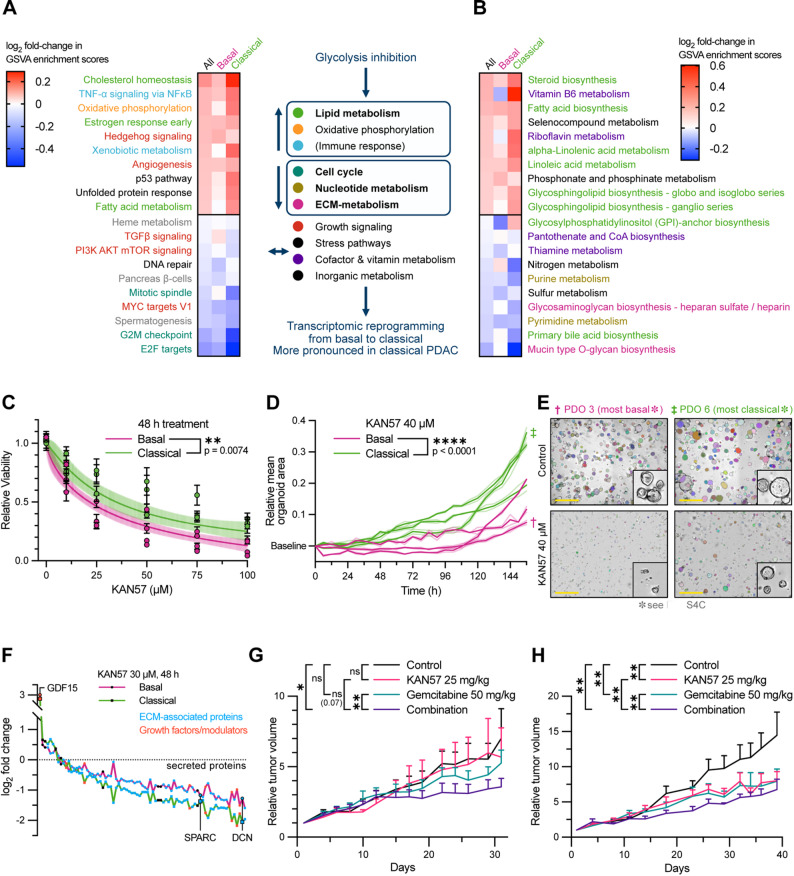
Targeting aggressive metabolic niches in PDAC through glycolysis inhibition. (**A**-**B**) Heatmap showing most enriched MSigDB Hallmark (**A**) and KEGG metabolism (**B**). Gene sets in the PDO cohort upon PFKFB3 inhibition by 40 µM KAN0438757 for 24 h, using gene set variation analysis. Data presented as log2 fold-changes derived from differential expression analysis of GSVA enrichment scores (untreated vs. treated PDOs). Pathways are grouped by functional categories (colored labels). Color scale: red, high expression; blue, low expression. (**C**) Cell viability of basal (BxPC-3, MIA PaCa-2, PANC-1, PaTu 8988T) and classical (Capan-1, Capan-2, HPAF II) PDAC cell lines treated with glycolysis inhibitor KAN0438757 for 48 h. Viability was calculated relative to the vehicle-treated control cells (means ± SD). Curves per cell lines and subtype (shaded area, 95% CI) fitted to variably sloped inhibitor vs. response model. Two-way ANOVA with Šidák correction. (**D**) IncuCyte real-time proliferation assay of basal and classical PDO lines (n=6) treated with 40 µM KAN0438757 for up to 156 h. PDOs counted and measured using the OrganoID algorithm. Areas normalized to treatment start and to vehicle-control at the end of the experiment (area of the vehicle-treated control organoids at t=156 h defined as 1.0 per PDO line). Curves fitted to a logistic growth model with Y0 constrained to 1. Two-way ANOVA (see also Fig. S7B for total area). (**E**) Representative images of basal and classical PDOs treated with 40 µM KAN0438757 at the end of treatment with overlayed OrganoID detection mask. Scale bars: 800 µm. (**F**) Secretome analysis of basal (n=3) and classical (n=4) PDAC PDO co-cultures treated with 30 µM KAN0438757 for 48 h. Dot plot shows differentially secreted proteins ranked by log2 fold-change upon treatment relative to control. Blue dots indicate ECM-associated proteins; red dots indicate growth factors or modulators. Color scale: pink, basal; green, classical. (**G**-**H**) Relative tumor volumes from basal (**G**) and classical (**H**) PDXs under single and combination therapy with KAN0438757 and/or gemcitabine in NRG mice cohorts. Treatment scheme in Fig. S7G. Data are mean ± SEM

Thus, we first confirmed the efficacy of glycolysis inhibition by metabolomic profiling of PDOs (*n* = 8) treated with 25 µM KAN0438757 for 24 h, demonstrating a consistent reduction in glycolytic and TCA cycle metabolites (Fig. S7A). Transcriptomic profiling of PDOs following glycolysis inhibition suggested a reprogramming towards the classical subtype, indicated by enrichment in all lipid metabolism-related hallmark gene sets (Fig. 6A). Additionally, there was a downregulation of basal-associated cell-cycle related gene sets (Myc targets, mitotic spindle, G2M checkpoint, and E2F targets) and enrichment in oxidative phosphorylation, suggesting increased mitochondrial activity to compensate for reduced glycolysis (Figs. 6A, S7A and Table 7). KEGG pathway analysis of PDOs transcriptomics after glycolysis inhibition confirmed this metabolic shift, showing enriched pathways associated with lipid metabolism and amino acid metabolism (Fig. 6B). Contrary to oxidative phosphorylation, nucleotide precursor and ECM-related metabolism decreased, indicating selective reprogramming of basal subtype signaling by glycolysis inhibition (Fig. 6B).

In line with these transcriptional shifts, inhibition of glycolysis by KAN0438757 in PDAC cell lines with defined subtypes resulted in reduced cell viability in both subtypes, with greater susceptibility in basal lines across various concentrations and time points (Fig. 6C). This was further supported by real-time proliferation measurements of classical and basal PDOs showing reduced proliferation upon treatment, with the basal PDOs being significantly more affected (Figs. 6D-E and S7B).

Overall, our results demonstrate that pharmacological inhibition of glycolysis via PFKFB3 inhibition reduced viability across subtype‑defined PDAC cell lines and PDOs from both subtypes, with basal models exhibiting the greatest sensitivity.

### Glycolysis inhibition reduces PDAC stromal permissiveness

Changes in metabolism not only directly affect tumor cells but also reshape the entire tumor microenvironment, altering stromal behavior and matrix composition [97, 98]. Therefore, to explore the potential consequences of glycolysis targeting on the tumor-stroma crosstalk and delineate the stromal response to glycolytic blockade, we performed mass-spectrometry-based proteomic profiling of conditioned media from PDO co-cultures with human fibroblasts and endothelial cells. KAN0438757 treatment of subtype‑defined PDOs (Fig. S7C) significantly lowered the secretion of key ECM proteins (Fig. 6F and Table S6). Specifically, KAN0438757 significantly reprogrammed the secretome, most notably reducing collagen isoforms such as type I to V, and other core ECM components such as glycoproteins (e.g. FN1, FBN1, SPARC, LAMC2, CTHRC1) [99–103], proteoglycans (e.g. SPOCK1 [104]), and ECM-associated growth factors (e.g. ANGPTL4, GAS6) [105, 106], all proteins with direct or indirect pro-tumorigenic stromal functions (Fig. 6F). Interestingly, SPARC and DCN, both identified among top-ranking marker genes for fibroblasts in meta-analysis (Figs. 2B and S3C, Table S4), also appeared affected by glycolysis inhibition, reinforcing their relevance to the PDAC tumor microenvironment. Moreover, these alterations are in line with the signaling changes induced by glycolytic inhibition, where ECM-related pathways are among the most downregulated metabolism gene sets (Fig. 6A), and were particularly pronounced in the lipogenic-classical subgroup, consistent with its more glandular and well-differentiated histology and correlating with a more active secretory program. Of note, the gene encoding GDF-15, by far the most induced protein upon glycolysis inhibition, was previously identified as a positive regulator of PFKFB3 in PDAC cancer cells (Fig. S6E), suggesting a compensatory feedback behavior.

In sum, these data indicate the critical role of glycolytic flux in sustaining PDAC‑associated matrix production, as ECM synthesis is a metabolically demanding process [107]; glycolysis blockade therefore disrupts tumor‑driven ECM deposition and may weaken the structural and biochemical support that fosters tumor progression and microenvironment permissiveness.

### Combination treatment via glycolysis inhibition reduces tumor growth in a subtype-independent manner

To further explore the results obtained via glycolysis inhibition beyond PDO models, we performed in vivo combination treatment using KAN0438757 and the standard PDAC chemotherapy agent gemcitabine on patient-derived xenografts (PDX). Taking advantage of the PDAC PDX platform established at the Clinical Research Unitf 5002 (CRU 5002, Göttingen, Germany), we selected one basal and one classical PDX for in vivo evaluation based on expression of the established molecular subtype markers GATA6 and p63 [108], and on RNA-sequencing data generated from earlier passages of the same PDX models (Figs. S7D–F; Table S7). When an average tumor volume of 100–200 mm^3^ was reached, mice were randomized into four groups: (i) control - sham; (ii) KAN0438757 25 mg/kg; (iii) gemcitabine 50 mg/kg, and (iv) combination: KAN0438757 + gemcitabine. Treatment was conducted for approximately five weeks, and tumors harvested 24 h after the last intervention (Fig. S7G). While single-treated mice did not show significant clinical alterations, a reduction in body weight could be observed in both subtypes, especially in the combination cohort (Figs. S7H-I). Additionally, dehydration was observed in both cohorts; however, in the basal PDX cohort the signs progressed to severe dehydration, requiring euthanasia approximately one week earlier, in accordance with the study’s predefined humane endpoint guidelines. Surprisingly, while single-treatment with KAN0438757 did not affect tumor growth in the basal PDX, the classical PDX showed a significant reduction in volume. Importantly, in both cohorts, combination therapy clearly reduced tumor volumes (Figs. 6G-H), reinforcing that pharmacological inhibition of glycolysis via PFKFB3 inhibition might be a potential therapy target for PDAC patients independent of the patient’s cancer cell subtype.

## Discussion

PDAC remains among the deadliest malignancies, largely because of its pronounced genetic, metabolic, and micro-environmental heterogeneity. In this work, spatially-resolved transcriptomics of resected PDACs allowed us to assemble a molecular platform that unifies PDAC epithelial, stromal, as well as normal pancreatic parenchymal compartments in their native context.

Building on recent advances in spatial and single-cell spatial transcriptomics, PDAC is now understood as a spatially-organized yet highly plastic ecosystem in which molecular subtypes coexist, intermix and adapt to local microenvironmental constraints [27, 28]. Previous studies have revealed conserved tumor–microenvironment architectures, invasion-associated niche programs and pronounced metabolic heterogeneity across primary and metastatic sites [24, 29]. However, how these spatial tumor states can be leveraged to inform therapeutic strategies remains largely unresolved, highlighting a key translational gap.

By integrating 42,035 spaRNA-seq spots with multiplexed protein validation, we uncovered a compact, pan-patient marker set that robustly demarcates major cell types. Among these, MLPH emerged as a tumor-restricted antigen that mirrors the distribution of the clinically explored glycoprotein MUC1, opening an immediate avenue for composite biomarker development. Throughout all patients, cancer cell clusters exhibited enriched proliferative and metabolic programs, highlighting highly proliferative PDAC cells with distinct glycolytic or lipid metabolic states. Surprisingly, the “KRAS signaling low” transcriptional signature emerged as upregulated in cancer cells, at this point most likely reflecting context-dependent regulation of KRAS pathway output rather than potential cell-type misclassification [109, 110].

Within the desmoplastic stromal niche, our data provide confirmation that myofibroblastic and inflammatory cancer-associated fibroblasts coexist, governed by *FN1*-*SPARC*-*DCN* ECM programs on one side and IL6/JAK/STAT-skewed secretomes on the other. Both programs converge on immuno-evasive *SPP1*-*CD44* and *LGALS9*-*CD44* circuits, likely reinforcing the immunosuppressive phenotype of PDAC, in line with previous studies [68, 72, 73, 103, 111].

Notably, an integrated multi-modal transcriptomic study reported coordinated upregulation of immune-related signaling programs in fibroblasts, including IL2/STAT5 and IL6/JAK/STAT3, supporting a conserved role for CAFs in stromal–immune crosstalk and immune modulation. In that study, a POSTN⁺ fibroblast subset spatially co-localized with SPP1⁺ macrophages, consistent with a coordinated stromal–immune axis associated with aggressive tumor organization and disease progression [112]. Complementary single-cell and spatial analyses further defined an extracellular matrix-remodeling CAF subtype engaging tumor epithelial cells via the POSTN–ITGAV/ITGB5 axis, activating PI3K/AKT/β-catenin signaling and promoting EMT [113].

While recent studies focused on finer CAF subtyping, our dataset captures a robust, collective CAF signature and CAF–tumor interactions across the PDAC stroma. Within this framework, growth factor and immunosuppressive signaling axes emerge as key, collectively relevant CAF functions, contextualizing CAF subtype-specific observations in a broader microenvironmental landscape.

Most current approaches to classifying PDAC rely on bulk multi-gene transcriptional profiles to stratify tumors into defined categories, with the prevailing consensus distinguishing between classical and basal subtypes. Our data support the notion that basal and classical programs represent distinct biological states within pancreatic cancer. Basal-like regions display transcriptional features associated with aggressive behavior, including increased proliferation, EMT and invasiveness, whereas classical cancer cells retain traits of a more differentiated state, such as endocrine signaling and lipid metabolism. Importantly, despite this subtype-specific heterogeneity, cancer cells across patients consistently maintain a shared malignant transcriptional identity that is clearly distinct from CAFs, underscoring the stability of cancer cell–intrinsic programs across tumors.

Emerging data, however, indicate that the dichotomy of basal and classical subtypes rather represents a spectrum of transcriptional states, with mixed phenotypes, accompanied by complex tumor heterogeneity, which is still poorly understood [31]. Our work provides spatially-resolved intra-tumoral subtyping based on a multi-gene transcriptional signature, thereby expanding the resources available for in-depth characterization of designated classical and basal areas within the tumor. Building on this framework, we were able to perform trajectory inference analysis on spatially resolved cancer cell signatures, revealing that the canonical classical and basal subtypes are not fixed entities but rather opposing poles of a transcriptional continuum. Classical regions, enriched for lipid-handling pathways and early differentiation markers (e.g., *HNF4A*,* GPX2*), cluster at the origin of pseudotime, whereas basal regions, distinguished by glycolytic and cell-cycle programs, invasive front localization and common aggressiveness markers (e.g.,* KRT17*,* VIM*,* PTHLH*), reside at the terminus. This gradual shift couples epithelial plasticity to metabolic rewiring, pinpointing glycolysis as a non-redundant driver of late, therapy-refractory tumor states and supporting the hypothesis that the classical molecular phenotype constitutes the default pathway of pancreatic tumorigenesis, while acquisition of the basal phenotype occurs in a subset of tumors and serves as a hallmark of advanced progression [15]. Notably, we found small high-grade, metabolically active basal foci embedded within both classical and basal PDACs, challenging the dichotomous bulk-sequencing-derived approach of PDAC subtyping. We suggest these niches – characterized by increased proliferation, glycolysis and lipid metabolism, hypoxia and loss of epithelial differentiation markers – as potential drivers of cancer progression, especially when missed by conventional treatment. Moreover, we hypothesize that when located at the invasive fronts, these niches may drive invasion and metastatic dissemination.

Interestingly, in a recent study Hao Wu and colleagues [114] detected spatial metabolic heterogeneity within PDAC cancer cell areas, described as hypermetabolic and hypometabolic regions, which were defined by the expression of glycolysis/gluconeogenesis, pentose phosphate pathway, oxidative phosphorylation, and glutathione metabolisms [114]. However, setting metabolically high niches in context with spatial distribution of the classical and basal subtype is a new aspect of our study. In our dataset, these metabolic high niches are further characterized by activation of hypoxia-response programs and increased proliferative signatures, suggesting coordinated metabolic and microenvironmental adaptation. Although prior work has linked hypoxia to enhanced proliferative capacity in PDAC models [115, 116], our data do not establish a causal effect of hypoxia on proliferation. Instead, it indicates that hypoxia-response transcriptional programs characterize and/or spatially co-localize with highly proliferative tumor states, underscoring the context-dependent complexity of this relationship.

Pharmacological inhibition of PFKFB3 with KAN0438757 confirmed glycolysis as a molecular subtype-agnostic target. In PDOs, blockade of glycolysis redirected basal cultures toward a classical, lipid-metabolism dependent state, attenuated proliferative gene programs, and increased responsiveness to gemcitabine. Secretome proteomics further showed that glycolysis fuels synthesis of collagens, fibronectin, and SPARC, suggesting that tumor glycolytic output metabolically underwrites desmoplastic matrix deposition. Corroborating these findings in vivo, combination therapy with KAN0438757 and gemcitabine reduced tumor growth in both basal and classical patient-derived xenografts, underscoring the subtype-agnostic potential of glycolytic blockade and demonstrating that its inhibition not only compromises tumor cell viability across molecular subtypes but also might attenuate stromal support by depleting tumor-promoting matrix constituents.

Importantly, despite validation across complementary approaches capturing tumor heterogeneity, evolution and tumor-stromal interactions, our work faces limitations. The spatial transcriptomic dataset is constrained by the Visium spot size (~ 50 μm), which captures multiple neighboring cells and limits resolution for interrogating single cell-cell communication. Future studies using higher-resolution spatial platforms may address this limitation. In addition, the PDO and PDX cohorts are restricted to surgically resectable tumors and modest sample sizes, limiting representation of advanced and metastatic disease and inter-patient heterogeneity. Finally, the limited in vivo tolerability of the combination regimen highlights the need for further pharmacokinetic and pharmacodynamic optimization and careful dose scheduling prior to clinical translation.

Collectively, our data reveal a mosaic tumor architecture in which small, high‑grade basal niches are embedded within PDAC cancer areas regardless of their subtype. Our study shows that PDAC cannot be adequately understood or treated through bulk, single-label molecular subtyping alone, since diagnostic material is typically limited and cannot reliably capture the full subtype composition of a patient’s tumor. Metabolic identity, spatial context, and tumor-stroma crosstalk form an inseparable triad that drives disease behavior. Therapeutically, the metabolically “hot” niches we mapped should take precedence over bulk RNA-sequencing-derived subtype calls when selecting therapy, much as even a small G3 fraction dictates histopathological grade. Failing to recognize and target these energetic strongholds leaves a reservoir of highly adaptable, drug-resistant cells poised to drive recurrence.

## Conclusion

In this study, we spatially-resolved PDAC subtype heterogeneity and evolution using a multigene transcriptional signature, revealing a mosaic architecture in which small, high-grade “hot” basal niches are embedded within tumors irrespective of their subtype. Our findings show that these niches can be targeted by glycolysis inhibition in a subtype-agnostic manner. Ultimately, this work demonstrates that PDAC cannot be adequately understood or treated by bulk, single-label molecular subtyping alone.

## Supplementary Information

Supplementary Material 1: Clinical data on patient cohorts and quality metrics on spatial RNA-Seq and MACSima imaging, related to Figure 1.

Supplementary Material 2: Expression of canonical marker genes in identified cell type clusters, related to Figure 1.

Supplementary Material 3: Congruent cell type marker genes in PDAC, related to Figure 1.

Supplementary Material 4: Ranked cell type signature gene lists, related to Figure 2.

Supplementary Material 5: Differential gene expression between basal and classical PDAC, related to Figure 3.

Supplementary Material 6: Differentially secreted proteins after glycolysis inhibition in basal and classical PDAC co-cultures, related to Figure 6.

Supplementary Material 7: GSVA gene set expression matrix, related to Figure 6.

Supplementary Material 8: Glycolytic and lipid metabolism signature used for gene expression in PDX samples, related to Figure S7F.

Supplementary Material 9: Figure S1. Patient-wise analyses of spatial transcriptomics dataset identifies tissue clusters (Patients 1-9), related to Figure 1.

Supplementary Material 10: Figure S2. Patient-wise analyses of spatial transcriptomics dataset tissue clusters (Patients 10-14), related to Figure 1.

Supplementary Material 11: Figure S3. Gene signature meta-analysis identifies congruent cell type marker genes validated by multiplexed immunostaining, related to Figure 1.

Supplementary Material 12: Figure S4. Supplemental details on intra-tumoral heterogeneity, subtype plasticity and patient-level metabolism of PDAC cancer cells, related to Figure 4 and 5.

Supplementary Material 13: Figure S6. Highly active metabolic tumor niches define aggressive regions across PDAC subtypes (additional observations), related to Figure 5.

Supplementary Material 14: Figure S7. Additional in vitro and in vivo experiments substantiate findings, related to Figure 6.

## Data Availability

Any additional information required to reanalyze the data reported in this paper is available from the leading contacts upon request. The gene expression matrices of the data sets supporting the conclusions of this article are available under the Gene expression Omnibus website using the following login details: PDO - subtyping GSE309669: ubmruewkvlqxhyz https://www.ncbi.nlm.nih.gov/geo/query/acc.cgi?acc=GSE309669. PDOs - treated with KAN0438757, GSE309739: qzepuawqvnyvril https://www.ncbi.nlm.nih.gov/geo/query/acc.cgi. PDX GSE316631: ohytesokrjgrlgf https://www.ncbi.nlm.nih.gov/geo/query/acc.cgi?acc=GSE316631. Regarding the spatial RNA-sequencing data, we have uploaded the SpaceRanger processed data in: ARRAYEXPRESS (TISSUES, FFPE). The spatial RNA sequencing of 14 primary formalin fixated paraffin embedded (FFPE) PDAC tissue slices from 14 patients without pretreatment has been assigned ArrayExpress accession E-MTAB-15702 and is accessible under: https://www.ebi.ac.uk/biostudies/ArrayExpress/studies/E-MTAB-15702?key=e610fb07-8c3e-4009-aff3-7fbaecadaf82. No custom code was generated in this study. Bioinformatic analyses were performed using the proprietary web-based platform UniApp (Unicle Biomedical Data Science, Belgium, https://unicle.com/), which is available under a commercial license. The underlying source code is not publicly accessible. Tool version, analysis parameters, and input data are described in the methods and supplementary materials section to enable reproducibility.

## Abbreviations

PDAC: Pancreatic Ductal Adenocarcinoma
spaRNA-seq: Spatial RNA sequencing
FFPE: Formalin Fixed, Paraffin Embedded
PCA: Principal Component Analysis
UMAP: Uniform Manifold Approximation and Projection
KEGG: Kyoto Encyclopedia of Genes and Genomes
ECGM2: Endothelial Cell Growth Medium Type 2
H&E: Hematoxylin and Eosin
PFKFB3: 6-phosphofructo-2-kinase/fructose-2,6-bisphosphatase 3
UPLC: Ultra Performance Liquid Chromatography
PDX/PDO: Patient-derived Xenograft/Organoid
ECM: Extracellular Matrix
NES: Normalized Enrichment Score
MsigDB: Molecular Signatures Database
TCGA: The Cancer Genome Atlas

## Data availability

This study did not generate new unique reagents.
