# Supplementary figures and images for: Spatially-resolved subtype progression reveals metabolic vulnerabilities in pancreatic ductal adenocarcinoma
Source: Mol Cancer. 2026 Mar 27;25:112. doi: 10.1186/s12943-026-02628-3 (PMC13088643; doi:10.1186/s12943-026-02628-3)

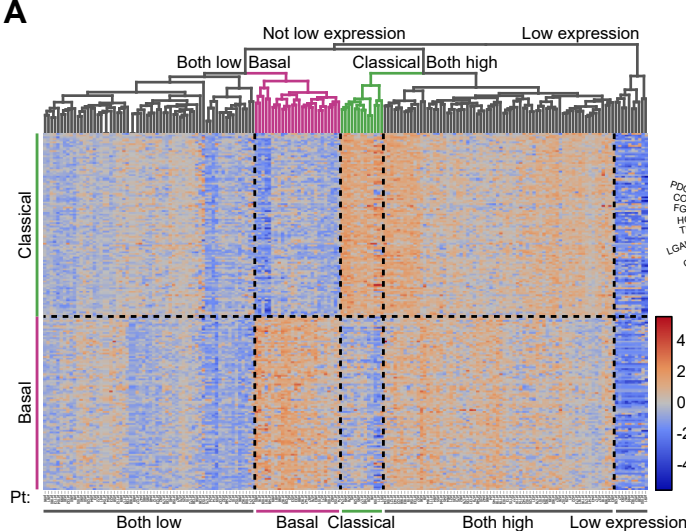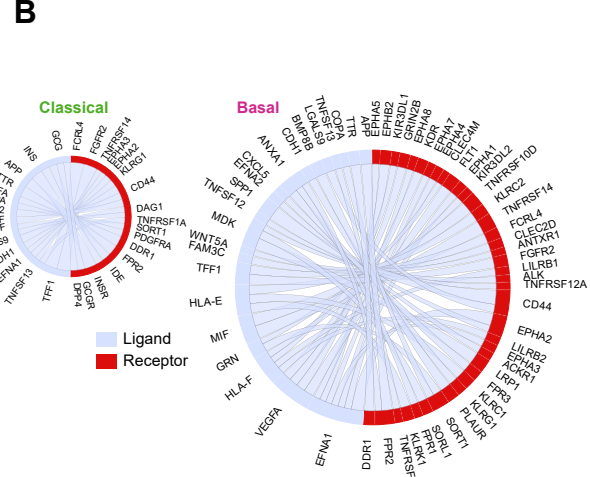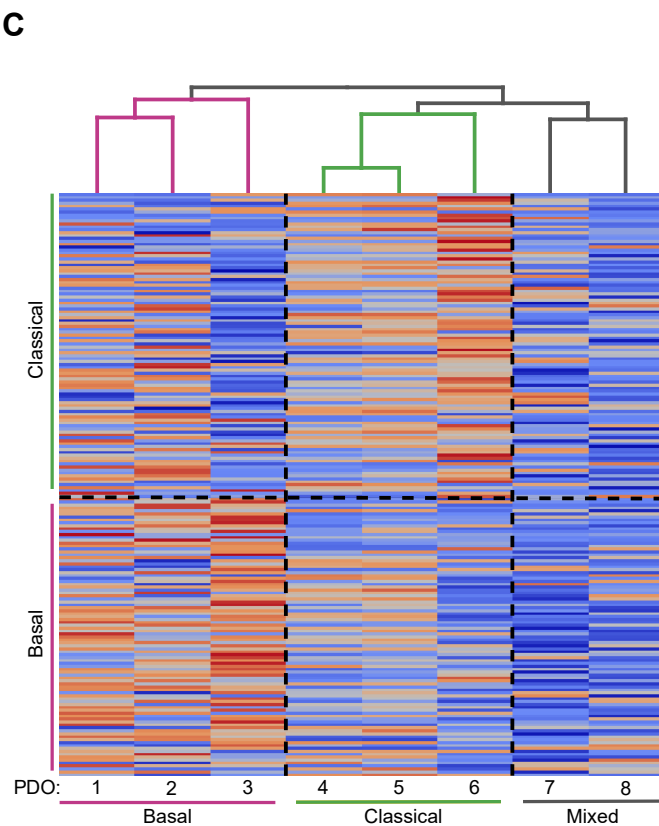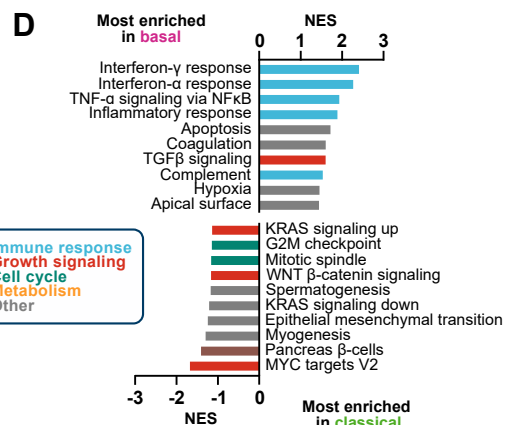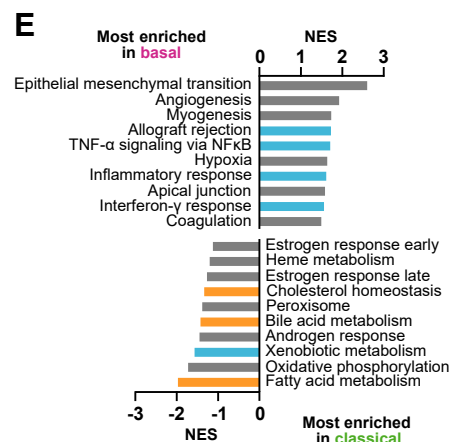

Supplement: Supplementary file 12 — Supplementary Material 12: Figure S4. Supplemental details on intra-tumoral heterogeneity, subtype plasticity and patient-level metabolism of PDAC cancer cells, related to Figure 4 and 5. [file 12943_2026_2628_MOESM12_ESM.pdf]

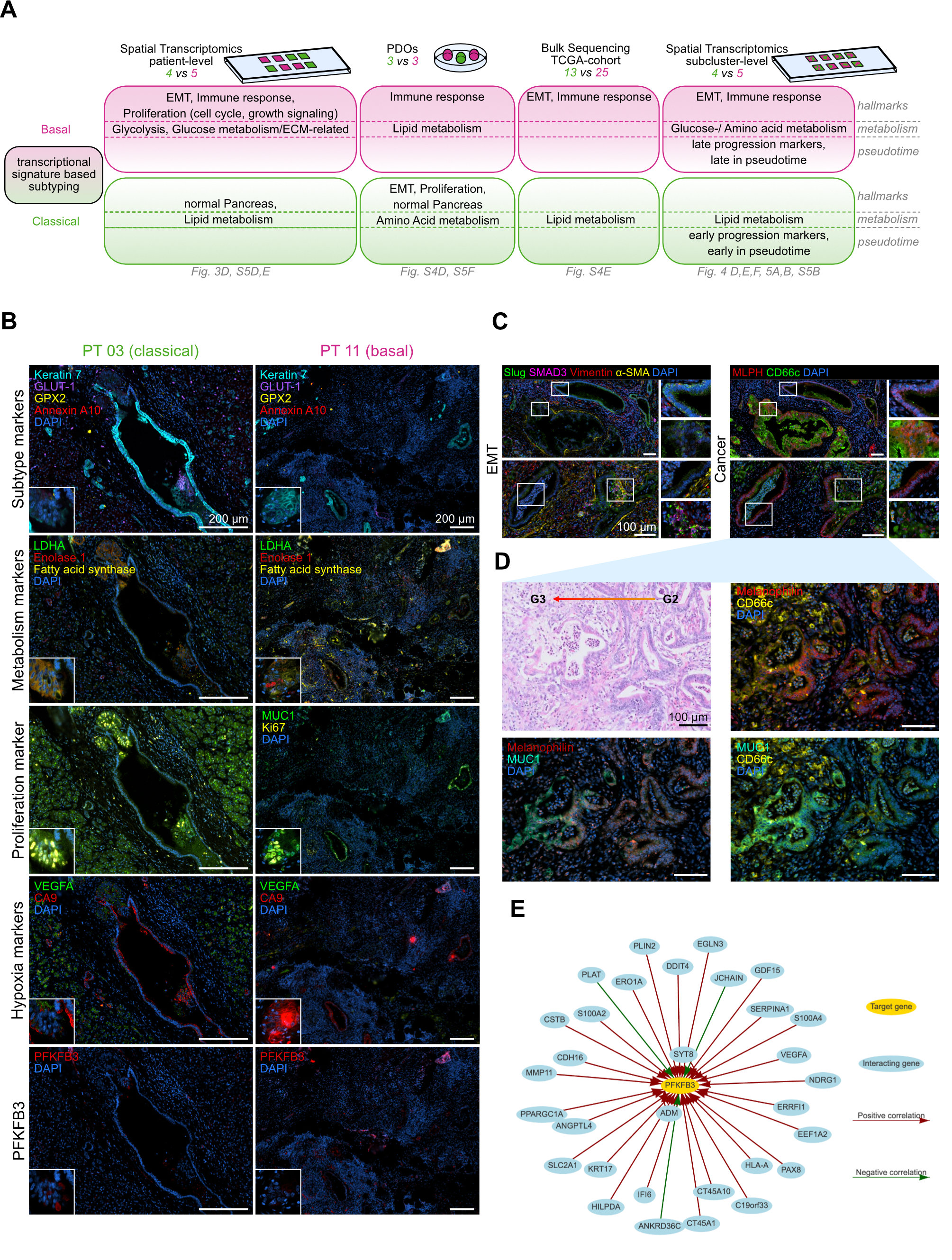

Supplement: Supplementary file 13 — Supplementary Material 13: Figure S6. Highly active metabolic tumor niches define aggressive regions across PDAC subtypes (additional observations), related to Figure 5. [file 12943_2026_2628_MOESM13_ESM.jpg]
